# Supplementary material for: Genomic epidemiology reveals transmission patterns and dynamics of SARS-CoV-2 in Aotearoa New Zealand
Source: Nat Commun. 2020 Dec 11;11:6351. doi: 10.1038/s41467-020-20235-8 (PMC7733492; doi:10.1038/s41467-020-20235-8)
Supplement: Supplementary file 2 — Descriptions of Additional Supplementary Files [file 41467_2020_20235_MOESM2_ESM.pdf]

Descriptions of Additional Supplementary Files

Supplementary Data 1

Description: A list of genomes and which amplification and sequencing method was used in for each case. A list of GISAID accession numbers from the global data set used in this study.
