# Supplementary material for: Genomic epidemiology reveals transmission patterns and dynamics of SARS-CoV-2 in Aotearoa New Zealand
Source: Nat Commun. 2020 Dec 11;11:6351. doi: 10.1038/s41467-020-20235-8 (PMC7733492; doi:10.1038/s41467-020-20235-8)
Supplement: Supplementary file 3 — Supplementary Data 1 [file 41467_2020_20235_MOESM3_ESM.pdf]

Isolate (GISAID accession number)	Sequencing protocol
20VR0189	articV1
20VR0195	articV1
20VR0206	articV1
20VR0275	articV1
20VR0276	articV1
20VR0334	articV1
20VR0461	articV1
20VR0635	articV1
20VR0174	articV1
20VR0759	articV3
20VR0760	articV3
20VR0766	articV3
20VR0769	articV3
20VR0993	articV3
20VR0997	articV3
20VR1000	articV3
20VR1019	articV3
20VR1020	articV3
20VR1076	articV3
20VR1108	articV3
20VR1109	articV3
20VR1114	articV3
20VR1271	articV3
20VR1275	articV3
20VR1276	articV3
20VR1279	articV3
20VR1280	articV3
20VR1282	articV3
20VR1283	articV3
20VR1289	articV3
20VR1292	articV3
20VR1293	articV3
20VR1449	articV3
20VR1453	articV3
20VR1454	articV3
20VR1499	articV3
20VR1616	articV3
20VR1617	articV3
20VR1619	articV3

20VR1620	articV3
20VR1621	articV3
20VR1622	articV3
20VR1623	articV3
20VR1625	articV3
20VR1626	articV3
20VR1627	articV3
20VR1759	articV3
20VR1760	articV3
20VR1765	articV3
20VR1769	articV3
20VR1771	articV3
20VR1774	articV3
20VR1777	articV3
20VR1778	articV3
20VR1781	articV3
20VR1783	articV3
20VR1784	articV3
20VR1791	articV3
20VR1803	articV3
20VR1804	articV3
20VR1805	articV3
20VR1824	articV3
20VR1829	articV3
20VR1831	articV3
20VR1836	articV3
20VR1839	articV3
20VR1841	articV3
20VR1847	articV3
20VR1850	articV3
20VR1865	articV3
20VR1893	articV3
20VR1894	articV3
20VR1896	articV3
20VR1899	articV3
20VR1900	articV3
20VR1911	articV3
20VR1915	articV3
20VR1927	articV3
20VR1928	articV3
20VR1929	articV3
20VR1930	articV3

20VR1933	articV3
20VR1935	articV3
20VR1945	articV3
20VR1947	articV3
20VR1950	articV3
20VR1951	articV3
20VR1952	articV3
20VR1954	articV3
20VR1955	articV3
20VR1957	articV3
20VR1962	articV3
20VR1963	articV3
20VR1978	articV3
20VR1980	articV3
20VR1981	articV3
20VR1984	articV3
20VR1985	articV3
20VR1986	articV3
20VR1987	articV3
20VR1992	articV3
20VR1995	articV3
20VR2001	articV3
20VR2005	articV3
20VR2010	articV3
20VR2011	articV3
20VR2012	articV3
20VR2019	articV3
20VR2020	articV3
20VR2022	articV3
20VR2024	articV3
20VR2025	articV3
20VR2038	articV3
20VR2041	articV3
20VR2042	articV3
20VR2046	articV3
20VR2048	articV3
20VR2050	articV3
20VR2052	articV3
20VR2053	articV3
20VR2054	articV3
20VR2055	articV3
20VR2056	articV3

20VR2060	articV3
20VR2062	articV3
20VR2066	articV3
20VR2071	articV3
20VR2072	articV3
20VR2080	articV3
20VR2082	articV3
20VR2083	articV3
20VR2089	articV3
20VR2091	articV3
20VR2098	articV3
20VR2106	articV3
20VR2108	articV3
20VR2109	articV3
20VR2472	articV3
20VR2473	articV3
20VR2477	articV3
20VR2478	articV3
20VR2479	articV3
20VR2481	articV3
20VR2483	articV3
20VR2484	articV3
20VR2487	articV3
20VR2493	articV3
20VR2499	articV3
20VR2500	articV3
20VR2504	articV3
20VR2505	articV3
20VR2506	articV3
20VR2516	articV3
20VR2518	articV3
20VR2521	articV3
20VR2524	articV3
20VR2525	articV3
20VR2528	articV3
20VR2529	articV3
20VR2531	articV3
20VR2532	articV3
20VR2534	articV3
20VR2536	articV3
20VR2537	articV3
20VR2540	articV3

20VR2541	articV3
20VR2550	articV3
20VR2559	articV3
20VR2562	articV3
20VR2563	articV3
20VR2572	articV3
20VR2584	articV3
20VR2585	articV3
20VR2586	articV3
20VR2647	articV3
20VR2648	articV3
20VR2649	articV3
20VR2650	articV3
20VR2651	articV3
20VR2652	articV3
20VR2653	articV3
20VR2654	articV3
20VR2656	articV3
20VR2657	articV3
20VR2659	articV3
20VR2660	articV3
20VR2662	articV3
20VR2663	articV3
20VR2666	articV3
20VR2667	articV3
20VR2669	articV3
20VR2670	articV3
20VR2671	articV3
20VR2711	articV3
20VR2712	articV3
20VR2713	articV3
20VR2714	articV3
20VR2715	articV3
20VR2716	articV3
20VR2717	articV3
20VR2718	articV3
20VR2719	articV3
20VR2720	articV3
20VR2721	articV3
20VR2722	articV3
20VR2723	articV3
20VR2724	articV3

20VR2726	articV3
20VR2727	articV3
20VR2730	articV3
20VR2731	articV3
20VR2732	articV3
20VR2733	articV3
20VR2918	articV3
20VR2919	articV3
20VR2920	articV3
20VR2922	articV3
20VR2923	articV3
20VR2924	articV3
20VR2925	articV3
20VR2926	articV3
20VR2928	articV3
20VR2929	articV3
20VR2930	articV3
20VR2933	articV3
20VR2934	articV3
20VR2935	articV3
20VR2936	articV3
20VR2937	articV3
20VR2940	articV3
20VR2942	articV3
20VR2943	articV3
20VR2944	articV3
20VR2946	articV3
20VR2947	articV3
20VR2948	articV3
20VR2949	articV3
20VR2950	articV3
20VR2951	articV3
20VR2952	articV3
20VR2953	articV3
20VR2955	articV3
20VR2956	articV3
20VR2957	articV3
20VR2958	articV3
20VR2959	articV3
20VR3006	articV3
20VR3007	articV3
20VR3009	articV3

20VR3012	articV3
20VR3016	articV3
20VR3017	articV3
20VR3024	articV3
20VR3025	articV3
20VR3026	articV3
20VR3027	articV3
20VR3029	articV3
20VR3030	articV3
20VR3031	articV3
20VR3032	articV3
20VR3033	articV3
20VR3034	articV3
20VR3036	articV3
20VR3037	articV3
20VR3038	articV3
20VR3040	articV3
20VR3044	articV3
20VR3056	articV3
20VR3060	articV3
20VR3061	articV3
20VR3062	articV3
20VR3063	articV3
20VR3066	articV3
20VR3068	articV3
20VR3070	articV3
20VR3071	articV3
20VR3076	articV3
20VR3084	articV3
20VR3088	articV3
20VR3089	articV3
20VR3096	articV3
20VR3098	articV3
20VR3099	articV3
20VR3112	articV3
20VR3128	articV3
20VR3135	articV3
20VR3137	articV3
20VR3139	articV3
20VR3140	articV3
20VR3141	articV3
20VR3142	articV3

20VR3144	articV3
20VR3145	articV3
20VR3146	articV3
20VR3147	articV3
20VR3151	articV3
20VR3152	articV3
20VR3156	articV3
20VR3157	articV3
20VR3159	articV3
20VR3160	articV3
20VR3161	articV3
20VR3162	articV3
20VR3165	articV3
20VR3167	articV3
20VR3168	articV3
20VR3170	articV3
20VR3171	articV3
20VR3173	articV3
20VR3175	articV3
20VR3176	articV3
20VR3177	articV3
20VR3178	articV3
20VR3186	articV3
20VR3187	articV3
20VR3188	articV3
20VR3190	articV3
20VR3192	articV3
20VR3194	articV3
20VR3195	articV3
20VR3197	articV3
20VR3198	articV3
20VR3201	articV3
20VR3202	articV3
20VR3204	articV3
20VR3205	articV3
20VR3206	articV3
20VR3207	articV3
20VR3476	articV3
20VR3477	articV3
20VR3480	articV3
20VR3481	articV3
20VR3482	articV3

20VR3483	articV3
20VR3486	articV3
20VR3489	articV3
20VR3490	articV3
20VR3491	articV3
20VR3493	articV3
20VR0462	nsw
20VR0465	nsw
20VR0514	nsw
20VR0999	nsw
20VR0995	nsw
20VR0994	nsw
20VR0996	nsw
20VR0761	nsw
20VR0770	nsw
20VR0989	nsw
20VR3185	nsw
20VR1018	nsw
20VR3136	nsw
20VR0987	nsw
20VR1285	nsw
20VR1004	nsw
20VR1290	nsw
20VR1286	nsw
20VR0712	nsw
20VR1110	nsw
20VR1022	nsw
20VR3143	nsw
20VR1115	nsw
20VR1278	nsw
20VR1277	nsw
20VR0933	nsw
20VR0914	nsw
20VR0901	nsw
20VR1274	nsw
20VR1273	nsw
20VR1450	nsw
20VR1016	nsw
20VR1291	nsw
20VR1298	nsw
20VR1297	nsw
20VR1113	nsw

20VR1272	nsw
20VR1281	nsw
20VR1452	nsw
20VR3149	nsw
20VR1792	nsw
20VR1793	nsw
20VR1794	nsw
20VR1799	nsw
20VR1795	nsw
20VR1798	nsw
20VR1078	nsw
20VR1451	nsw
20VR3150	nsw
20VR2471	nsw
20VR3163	nsw
20VR1797	nsw
20VR2474	nsw
20VR2556	nsw
20VR2573	nsw
20VR1796	nsw
20VR2558	nsw
20VR1888	nsw
20VR3154	nsw
20VR2571	nsw
20VR3155	nsw
20VR1618	nsw
20VR2476	nsw
20VR2490	nsw
20VR2475	nsw
20VR2489	nsw
20VR2488	nsw
20VR2485	nsw
20VR2486	nsw
20VR2494	nsw
20VR2491	nsw
20VR2495	nsw
20VR1192	nsw
20VR2492	nsw
20VR2497	nsw
20VR2498	nsw
20VR3158	nsw
20VR1133	nsw

20VR1624	nsw
20VR1615	nsw
20VR3164	nsw
20VR2503	nsw
20VR2501	nsw
20VR2574	nsw
20VR2557	nsw
20VR3166	nsw
20VR1756	nsw
20VR2507	nsw
20VR1380	nsw
20VR2519	nsw
20VR2517	nsw
20VR2520	nsw
20VR2522	nsw
20VR1764	nsw
20VR1758	nsw
20VR3172	nsw
20VR3169	nsw
20VR1775	nsw
20VR2526	nsw
20VR2560	nsw
20VR1988	nsw
20VR2576	nsw
20VR2561	nsw
20VR2552	nsw
20VR3180	nsw
20VR1782	nsw
20VR1993	nsw
20VR2527	nsw
20VR1581	nsw
20VR2538	nsw
20VR2530	nsw
20VR2579	nsw
20VR2013	nsw
20VR1779	nsw
20VR1773	nsw
20VR2539	nsw
20VR2555	nsw
20VR1780	nsw
20VR1776	nsw
20VR2554	nsw

20VR1772	nsw
20VR2000	nsw
20VR2570	nsw
20VR1597	nsw
20VR3179	nsw
20VR3022	nsw
20VR3196	nsw
20VR1990	nsw
20VR3181	nsw
20VR3182	nsw
20VR1561	nsw
20VR2535	nsw
20VR1825	nsw
20VR1821	nsw
20VR1822	nsw
20VR1826	nsw
20VR1827	nsw
20VR1823	nsw
20VR3183	nsw
20VR1820	nsw
20VR1818	nsw
20VR1848	nsw
20VR1817	nsw
20VR1815	nsw
20VR1813	nsw
20VR1819	nsw
20VR1816	nsw
20VR1851	nsw
20VR1814	nsw
20VR1852	nsw
20VR1996	nsw
20VR1991	nsw
20VR1997	nsw
20VR1998	nsw
20VR1842	nsw
20VR2581	nsw
20VR3191	nsw
20VR2546	nsw
20VR2547	nsw
20VR2533	nsw
20VR2549	nsw
20VR1853	nsw

20VR2545	nsw
20VR1994	nsw
20VR2544	nsw
20VR1838	nsw
20VR1828	nsw
20VR1812	nsw
20VR1806	nsw
20VR1808	nsw
20VR1807	nsw
20VR1811	nsw
20VR1809	nsw
20VR1846	nsw
20VR2003	nsw
20VR1843	nsw
20VR1840	nsw
20VR1844	nsw
20VR1845	nsw
20VR1833	nsw
20VR1837	nsw
20VR3200	nsw
20VR2941	nsw
20VR2006	nsw
20VR2004	nsw
20VR1830	nsw
20VR1949	nsw
20VR2009	nsw
20VR1835	nsw
20VR1858	nsw
20VR2007	nsw
20VR1856	nsw
20VR1857	nsw
20VR2932	nsw
20VR3210	nsw
20VR3203	nsw
20VR2017	nsw
20VR2016	nsw
20VR3011	nsw
20VR2018	nsw
20VR1862	nsw
20VR1864	nsw
20VR3010	nsw
20VR1863	nsw

20VR1860	nsw
20VR1861	nsw
20VR1917	nsw
20VR1867	nsw
20VR1859	nsw
20VR1868	nsw
20VR2027	nsw
20VR2031	nsw
20VR2030	nsw
20VR2029	nsw
20VR2026	nsw
20VR2036	nsw
20VR1916	nsw
20VR1895	nsw
20VR1889	nsw
20VR1897	nsw
20VR1890	nsw
20VR1891	nsw
20VR3209	nsw
20VR3208	nsw
20VR1940	nsw
20VR1908	nsw
20VR1913	nsw
20VR1904	nsw
20VR1903	nsw
20VR1901	nsw
20VR1914	nsw
20VR1907	nsw
20VR1887	nsw
20VR2045	nsw
20VR1944	nsw
20VR2051	nsw
20VR1943	nsw
20VR2021	nsw
20VR2023	nsw
20VR2076	nsw
20VR2077	nsw
20VR2067	nsw
20VR2093	nsw
20VR1924	nsw
20VR1923	nsw
20VR2088	nsw

20VR2085	nsw
20VR3039	nsw
20VR1925	nsw
20VR2095	nsw
20VR1931	nsw
20VR2096	nsw
20VR1921	nsw
20VR2092	nsw
20VR1936	nsw
20VR1946	nsw
20VR2065	nsw
20VR1961	nsw
20VR1965	nsw
20VR3041	nsw
20VR2059	nsw
20VR1959	nsw
20VR1960	nsw
20VR2058	nsw
20VR1964	nsw
20VR1956	nsw
20VR2063	nsw
20VR2068	nsw
20VR2061	nsw
20VR1979	nsw
20VR1982	nsw
20VR3014	nsw
20VR2105	nsw
20VR2101	nsw
20VR2094	nsw
20VR2075	nsw
20VR2078	nsw
20VR2074	nsw
20VR2079	nsw
20VR2073	nsw
20VR3019	nsw
20VR2103	nsw
20VR2081	nsw
20VR3004	nsw
20VR3003	nsw
20VR3005	nsw
20VR3045	nsw
20VR3075	nsw

20VR3110	nsw
20VR3064	nsw
20VR3117	nsw
20VR3093	nsw
20VR0765	nsw
20VR0990	nsw
20VR1870	nsw
20VR1910	nsw
20VR1941	nsw
20VR1948	nsw
20VR1999	nsw
20VR2002	nsw
20VR2049	nsw
20VR2097	nsw
20VR2482	nsw
20VR2548	nsw
20VR3008	nsw
20VR3116	nsw
20VR3119	nsw
20VR3138	nsw
20VR2086	nsw
20VR3065	nsw

'Andalusia_COV001884_2020_Spain_A.2_2020.19722222222'
'Argentina_PAIS_A003_2020_Argentina_B.1_2020.23611111111'
'Australia_NSW06_2020_Australia_B.4_2020.16111111111'
'Australia_NSW123_2020_Australia_B.1_2020.22777777778'
'Australia_NSW169_2020_Australia_B_2020.23055555556'
'Australia_NSW171_2020_Australia_B.1.1_2020.22222222222'
'Australia_NSW183_2020_Australia_A.2_2020.22222222222'
'Australia_NSW203_2020_Australia_A.2_2020.225'
'Australia_NSW306_2020_Australia_B.1_2020.25'
'Australia_NSW333_2020_Australia_B.4_2020.22777777778'
'Australia_NSW338_2020_Australia_B.1.23_2020.26111111111'
'Australia_NSW34_2020_Australia_B.4_2020.19444444444'
'Australia_NSW36_2020_Australia_B.2.1_2020.19166666667'
'Australia_NSW500_2020_Australia_B.1_2020.28611111111'
'Australia_NSW506_2020_Australia_B.1.23_2020.24444444444'
'Australia_NSW532_2020_Australia_B.1_2020.35277777778'
'Australia_SAP012_2020_Australia_B.1.5_2020.25'
'Australia_SAP067_2020_Australia_B.1_2020.24444444444'
'Australia_SAP263_2020_Australia_A_2020.23333333333'
'Australia_SAP271_2020_Australia_B.1_2020.25'
'Australia_SAP294_2020_Australia_A.2_2020.23333333333'
'Australia_VIC1087_2020_Australia_A.1_2020.26388888889'
'Australia_VIC1139_2020_Australia_B.1_2020.26944444444'
'Australia_VIC1156_2020_Australia_B.1_2020.27222222222'
'Australia_VIC122_2020_Australia_B.1_2020.21944444444'
'Australia_VIC1271_2020_Australia_A.2_2020.28333333333'
'Australia_VIC1272_2020_Australia_B.1.1_2020.28333333333'
'Australia_VIC1490_2020_Australia_B.4_2020.32222222222'
'Australia_VIC1511_2020_Australia_B.1_2020.33333333333'
'Australia_VIC1567_2020_Australia_B.4_2020.34722222222'
'Australia_VIC1593_2020_Australia_B.1.36_2020.36111111111'
'Australia_VIC1638_2020_Australia_B.1_2020.33611111111'
'Australia_VIC1711_2020_Australia_B.1.1_2020.38333333333'
'Australia_VIC341_2020_Australia_B.1.5_2020.21944444444'
'Australia_VIC357_2020_Australia_B.1_2020.22222222222'
'Australia_VIC367_2020_Australia_A.2_2020.225'
'Australia_VIC373_2020_Australia_B_2020.225'
'Australia_VIC471_2020_Australia_A.1_2020.23611111111'
'Australia_VIC479_2020_Australia_B.1.23_2020.24166666667'
'Australia_VIC572_2020_Australia_B.2.1_2020.23333333333'
'Australia_VIC591_2020_Australia_B.3_2020.225'
'Australia_VIC679_2020_Australia_B.9_2020.23888888889'
'Australia_VIC681_2020_Australia_B.4_2020.23888888889'
'Australia_VIC684_2020_Australia_B.1_2020.24444444444'
'Australia_VIC774_2020_Australia_B.1.23_2020.21388888889'
'Australia_VIC784_2020_Australia_A.1.3_2020.24722222222'
'Australia_VIC884_2020_Australia_B.1.1_2020.26388888889'
'Australia_VIC88_2020_Australia_B.2.1_2020.20555555556'

'Australia_VIC893_2020_Australia_B.1.33_2020.26388888889'
'Australia_VIC929_2020_Australia_B.1_2020.24722222222'
'Australia_VIC95_2020_Australia_B.1_2020.20833333333'
'Austria_CeMM0146_2020_Austria_B.1.1.6_2020.15833333333'
'Austria_CeMM0170_2020_Austria_B.1.1.6_2020.18333333333'
'Austria_CeMM0266_2020_Austria_B.3_2020.23055555556'
'Bangladesh_BCSIR_NILMRC_069_2020_Bangladesh_B.1.36_2020.40277777778'
'Bangladesh_CHRF_0003_2020_Bangladesh_B.1.1_2020.29444444444'
'Bangladesh_CHRF_0010_2020_Bangladesh_B.1_2020.33888888889'
'Beijing_BJ556_2020_China_A_2020.09166666667'
'Beijing_BJ625_2020_China_B_2020.09722222222'
'Belgium_030959_2020_Belgium_B.1.1_2020.18888888889'
'Belgium_CD_030679_2020_Belgium_B.1.1_2020.18055555556'
'Belgium_CF_0327345_2020_Belgium_B.1.1_2020.23888888889'
'Belgium_DGL_0409415_2020_Belgium_B.2_2020.27222222222'
'Belgium_ITM_C340_2020_Belgium_B.1_2020.26388888889'
'Belgium_ITM_C366_2020_Belgium_B.1_2020.26944444444'
'Belgium_LJ_0325148_2020_Belgium_B.1.1.5_2020.23333333333'
'Belgium_MF_030546_2020_Belgium_B.1.1_2020.17777777778'
'Belgium_RR_0326315_2020_Belgium_B.1_2020.23611111111'
'Belgium_Rega_0328200_2020_Belgium_B.1.1_2020.24166666667'
'Belgium_SAA_0330367_2020_Belgium_B.1_2020.24722222222'
'Belgium_SC_0331378_2020_Belgium_B.1_2020.25'
'Belgium_ULG_10131_2020_Belgium_B.1.1.5_2020.26666666667'
'Belgium_VHV_0324118_2020_Belgium_B.2_2020.23055555556'
'Belgium_WCV_0408399_2020_Belgium_B.1.5_2020.26944444444'
'Belgium_WLM_0406275_2020_Belgium_B.1.1_2020.26388888889'
'Belgium_rega_0423297_2020_Belgium_B.3_2020.31111111111'
'BosniaandHerzegovina_ChVir7361_2020_Bosnia_and_Herzegovina_B.1.1_2020.23333333333'
'BosniaandHerzegovina_ChVir7367_2020_Bosnia_and_Herzegovina_B.1_2020.23888888889'
'Brazil_RJ_899_2020_Brazil_B.1.1_2020.24722222222'
'Brazil_SPBR_14_2020_Brazil_B.1.1_2020.17777777778'
'Brazil_SPBR_505_2020_Brazil_B.1.1_2020.28888888889'
'Canada_BC_6502001_2020_Canada_A.1_2020.19722222222'
'Canada_BC_78548_2020_Canada_B.2.1_2020.17777777778'
'Canada_NB_21_2020_Canada_B.1_2020.19722222222'
'Canada_ON_PHL3458_2020_Canada_B.1.1_2020.19722222222'
'Canada_ON_QGLO_01_2020_Canada_B.1.1_2020.21388888889'
'Canada_QC_AO9_2020_Canada_B.1_2020.27222222222'
'Canada_QC_AT6_2020_Canada_B.1_2020.26388888889'
'Chile_Santiago_48_2020_Chile_B.1_2020.22222222222'
'Chile_Santiago_53_2020_Chile_B.1.5_2020.25'
'Colombia_GVI97223_2020_Colombia_B.1.5_2020.26388888889'
'CzechRepublic_Seq5_2020_Czech_Republic_B.1.1_2020.21666666667'
'DRC_523_2020_Democratic_Republic_of_the_Congo_B.1.1.1_2020.24166666667'
'Denmark_ALAB_HH07_2020_Denmark_B.1_2020.19166666667'
'Denmark_ALAB_HH23_2020_Denmark_B.1_2020.19166666667'
'Denmark_ALAB_HH_170_2020_Denmark_B.1.1_2020.28888888889'

'Denmark_ALAB_SSI105_2020_Denmark_B.1_2020.18611111111'
'Denmark_ALAB_SSI150_2020_Denmark_B.1_2020.18888888889'
'Denmark_ALAB_SSI189_2020_Denmark_B.1_2020.18888888889'
'Denmark_ALAB_SSI219_2020_Denmark_B.1_2020.19166666667'
'Denmark_ALAB_SSI302_2020_Denmark_B.1_2020.19166666667'
'Denmark_ALAB_SSI377_2020_Denmark_B.1_2020.225'
'Denmark_ALAB_SSI_1102_2020_Denmark_B.1_2020.21388888889'
'Denmark_ALAB_SSI_1291_2020_Denmark_B.1_2020.25'
'Denmark_ALAB_SSI_245_2020_Denmark_B.1_2020.18055555556'
'Denmark_ALAB_SSI_676_2020_Denmark_B.1_2020.23888888889'
'Denmark_ALAB_SSI_692_2020_Denmark_B.1_2020.23888888889'
'Denmark_ALAB_SSI_831_2020_Denmark_B.1_2020.24722222222'
'Denmark_SSI_01_2020_Denmark_B.1.1_2020.15277777778'
'Egypt_CUNCI_HGC007_2_2020_Egypt_B.1_2020.33611111111'
'England_20099068804_2020_United_Kingdom_B.1.1_2020.16111111111'
'England_20099070104_2020_United_Kingdom_B.2_2020.16111111111'
'England_201000003_2020_United_Kingdom_B.2_2020.16666666667'
'England_20102003804_2020_United_Kingdom_B.2.1_2020.16944444444'
'England_20102004704_2020_United_Kingdom_B_2020.16944444444'
'England_201040110_2020_United_Kingdom_B.1_2020.16944444444'
'England_201040158_2020_United_Kingdom_B.2.1_2020.17222222222'
'England_201060055_2020_United_Kingdom_B.1_2020.175'
'England_201060066_2020_United_Kingdom_B.2_2020.175'
'England_201061457_2020_United_Kingdom_B.1.1_2020.16666666667'
'England_20106145903_2020_United_Kingdom_B.2_2020.17222222222'
'England_201080056_2020_United_Kingdom_B.1_2020.17777777778'
'England_201080113_2020_United_Kingdom_B.1_2020.17777777778'
'England_20108152504_2020_United_Kingdom_B.2_2020.17222222222'
'England_20109010304_2020_United_Kingdom_B.2.1_2020.18055555556'
'England_20109052506_2020_United_Kingdom_B_2020.18333333333'
'England_20109056906_2020_United_Kingdom_B.2.1_2020.17777777778'
'England_20109058906_2020_United_Kingdom_B_2020.18055555556'
'England_201090782_2020_United_Kingdom_B_2020.18055555556'
'England_201101382_2020_United_Kingdom_B.3_2020.18888888889'
'England_201140062_2020_United_Kingdom_B.1_2020.19444444444'
'England_201140338_2020_United_Kingdom_B.1_2020.18888888889'
'England_201161230_2020_United_Kingdom_B.2_2020.19166666667'
'England_201161424_2020_United_Kingdom_B.1.13_2020.19722222222'
'England_201161480_2020_United_Kingdom_B.1_2020.19444444444'
'England_201161565_2020_United_Kingdom_B.1.1_2020.19444444444'
'England_20118044507_2020_United_Kingdom_B.2.1_2020.2'
'England_20118050804_2020_United_Kingdom_B.1.1_2020.2'
'England_20118060904_2020_United_Kingdom_B.2.1_2020.2'
'England_20118133304_2020_United_Kingdom_B.2_2020.2'
'England_20118137803_2020_United_Kingdom_B_2020.19722222222'
'England_20119008704_2020_United_Kingdom_B.1.1_2020.19722222222'
'England_20119029804_2020_United_Kingdom_B.1_2020.19722222222'
'England_20119059404_2020_United_Kingdom_B.1.1_2020.19722222222'

'England_20119080704_2020_United_Kingdom_B.2.1_2020.2'
'England_20119092004_2020_United_Kingdom_B.2.1_2020.19444444444'
'England_20120001304_2020_United_Kingdom_B.1.1_2020.20833333333'
'England_20124009602_2020_United_Kingdom_B_2020.21388888889'
'England_20124034702_2020_United_Kingdom_B.2.1_2020.20833333333'
'England_20124097004_2020_United_Kingdom_B.2.5_2020.2'
'England_20126103304_2020_United_Kingdom_B.1_2020.21388888889'
'England_20129042806_2020_United_Kingdom_B.1.1_2020.19444444444'
'England_20129142004_2020_United_Kingdom_B_2020.19722222222'
'England_20129161204_2020_United_Kingdom_B_2020.19722222222'
'England_20130051604_2020_United_Kingdom_B.2.1_2020.225'
'England_20132004704_2020_United_Kingdom_B.1_2020.22777777778'
'England_20132023104_2020_United_Kingdom_B.2.1_2020.225'
'England_20132024004_2020_United_Kingdom_B.1.1_2020.22777777778'
'England_20132035604_2020_United_Kingdom_B.2.1_2020.23055555556'
'England_20132050802_2020_United_Kingdom_B.1_2020.225'
'England_20132107704_2020_United_Kingdom_B.2.1_2020.23055555556'
'England_20134010002_2020_United_Kingdom_B.2.1_2020.22777777778'
'England_20134028004_2020_United_Kingdom_B.1.1_2020.22777777778'
'England_20134077904_2020_United_Kingdom_B.1.1_2020.23055555556'
'England_201360130_2020_United_Kingdom_B.1_2020.23333333333'
'England_201380042_2020_United_Kingdom_B.1.1_2020.23333333333'
'England_201380049_2020_United_Kingdom_B.2.1_2020.23333333333'
'England_201380062_2020_United_Kingdom_B.1.1_2020.23333333333'
'England_20138019104_2020_United_Kingdom_B.1.1.7_2020.23611111111'
'England_20139001604_2020_United_Kingdom_B.2.1_2020.23888888889'
'England_20139022704_2020_United_Kingdom_B.1.1_2020.23888888889'
'England_20139030304_2020_United_Kingdom_B.1.13_2020.23611111111'
'England_20139058304_2020_United_Kingdom_B.1.13_2020.23888888889'
'England_20139064704_2020_United_Kingdom_B.2.1_2020.24166666667'
'England_20140007202_2020_United_Kingdom_B.1_2020.24166666667'
'England_20142026704_2020_United_Kingdom_B.1.1_2020.24444444444'
'England_20144036004_2020_United_Kingdom_B.1_2020.25'
'England_20146011904_2020_United_Kingdom_B.1_2020.25'
'England_20148002204_2020_United_Kingdom_B.3_2020.25277777778'
'England_20148048004_2020_United_Kingdom_B.2.1_2020.25277777778'
'England_20154074604_2020_United_Kingdom_B.2.1_2020.26666666667'
'England_20156047304_2020_United_Kingdom_B.1.5.4_2020.26388888889'
'England_201590147_2020_United_Kingdom_B.1.1_2020.27777777778'
'England_201640075_2020_United_Kingdom_B.1.1_2020.28611111111'
'England_20166054304_2020_United_Kingdom_B.1.1_2020.28333333333'
'England_20166067404_2020_United_Kingdom_B.2.1_2020.29166666667'
'England_20169002204_2020_United_Kingdom_B.1.1_2020.29444444444'
'England_20169030304_2020_United_Kingdom_B.1.1_2020.28888888889'
'England_20170048604_2020_United_Kingdom_B.1.1_2020.30277777778'
'England_20172002504_2020_United_Kingdom_B.1.1.7_2020.3'
'England_20172056804_2020_United_Kingdom_B.1.1_2020.30277777778'
'England_20186079504_2020_United_Kingdom_B.1.1_2020.325'

'England_20189176701_2020_United_Kingdom_B.1.1_2020.33333333333'
'England_20192018201_2020_United_Kingdom_B.1_2020.34166666667'
'England_20194106101_2020_United_Kingdom_B.1.1_2020.34444444444'
'England_20206026604_2020_United_Kingdom_B.1.36_2020.36111111111'
'England_BIRM_5E8CF_2020_United_Kingdom_B.1.1.1_2020.22777777778'
'England_BRIS_121B07_2020_United_Kingdom_B.1.1_2020.21388888889'
'England_BRIS_121C5F_2020_United_Kingdom_B.2.1_2020.21111111111'
'England_BRIS_121F29_2020_United_Kingdom_B.1.72_2020.21666666667'
'England_BRIS_12308E_2020_United_Kingdom_B.1.1_2020.26111111111'
'England_BRIS_1232C4_2020_United_Kingdom_B.1.1_2020.26111111111'
'England_BRIS_1238FF_2020_United_Kingdom_B.1_2020.26388888889'
'England_BRIS_123A53_2020_United_Kingdom_B.2.1_2020.26666666667'
'England_BRIS_123ACC_2020_United_Kingdom_B.1.1_2020.26666666667'
'England_BRIS_123BE7_2020_United_Kingdom_B.1.5_2020.26388888889'
'England_BRIS_123C3F_2020_United_Kingdom_B.1.1.1_2020.26666666667'
'England_BRIS_1247F1_2020_United_Kingdom_B_2020.23333333333'
'England_BRIS_1249EC_2020_United_Kingdom_B.1_2020.23333333333'
'England_BRIS_124B40_2020_United_Kingdom_B_2020.23333333333'
'England_BRIS_124CB6_2020_United_Kingdom_B.1.1_2020.23611111111'
'England_BRIS_1251D4_2020_United_Kingdom_B.1.1_2020.23888888889'
'England_BRIS_1255BA_2020_United_Kingdom_B.1_2020.23888888889'
'England_BRIS_125A8E_2020_United_Kingdom_B.2.2_2020.24444444444'
'England_BRIS_125CB5_2020_United_Kingdom_B.1_2020.24722222222'
'England_BRIS_125E0A_2020_United_Kingdom_B_2020.24722222222'
'England_BRIS_125ECE_2020_United_Kingdom_B.1_2020.24722222222'
'England_BRIS_12623A_2020_United_Kingdom_B.1.1_2020.275'
'England_BRIS_1262FE_2020_United_Kingdom_B.1.1.10_2020.275'
'England_BRIS_12649E_2020_United_Kingdom_B.1.1_2020.275'
'England_BRIS_12665C_2020_United_Kingdom_B.1.1_2020.27222222222'
'England_BRIS_126D57_2020_United_Kingdom_B.1.72_2020.26944444444'
'England_BRIS_128DDD_2020_United_Kingdom_B.1_2020.28611111111'
'England_BRIS_129D45_2020_United_Kingdom_B.1.5_2020.28888888889'
'England_BRIS_129E7F_2020_United_Kingdom_B.3_2020.29166666667'
'England_BRIS_12A38E_2020_United_Kingdom_B.1_2020.33055555556'
'England_BRIS_12A6B2_2020_United_Kingdom_B.1.1_2020.33055555556'
'England_BRIS_12AA10_2020_United_Kingdom_B_2020.33055555556'
'England_CAMB_1AAEB2_2020_United_Kingdom_B.1.1_2020.29444444444'
'England_CAMB_1AC375_2020_United_Kingdom_B.1_2020.32777777778'
'England_CAMB_1ADB5E_2020_United_Kingdom_B.1.1_2020.33888888889'
'England_CAMB_1ADBD6_2020_United_Kingdom_B.1_2020.33333333333'
'England_CAMB_1ADDEF_2020_United_Kingdom_B.1.1_2020.31944444444'
'England_CAMB_1AE531_2020_United_Kingdom_B.1.35_2020.325'
'England_CAMB_1AF1B4_2020_United_Kingdom_B.1.1_2020.32777777778'
'England_CAMB_1AF600_2020_United_Kingdom_B.1.5_2020.34722222222'
'England_CAMB_1AFA13_2020_United_Kingdom_B.1.30_2020.33611111111'
'England_CAMB_1AFCD1_2020_United_Kingdom_B.1_2020.37222222222'
'England_CAMB_1B00FF_2020_United_Kingdom_B.1.1_2020.35'
'England_CAMB_1B048A_2020_United_Kingdom_B.1.1_2020.33333333333'

'England_CAMB_1B09D6_2020_United_Kingdom_B.1.1_2020.36388888889'
'England_CAMB_1B1735_2020_United_Kingdom_B.1.1_2020.35277777778'
'England_CAMB_1B19A8_2020_United_Kingdom_B.1.5_2020.36944444444'
'England_CAMB_1B1C90_2020_United_Kingdom_B.1_2020.35833333333'
'England_CAMB_71AA2_2020_United_Kingdom_B.2.1_2020.25'
'England_CAMB_71E3D_2020_United_Kingdom_B.2_2020.24444444444'
'England_CAMB_71EF1_2020_United_Kingdom_B_2020.24444444444'
'England_CAMB_71F0D_2020_United_Kingdom_B.1.1_2020.24444444444'
'England_CAMB_71FB2_2020_United_Kingdom_B.1_2020.24166666667'
'England_CAMB_722E5_2020_United_Kingdom_B.2.1_2020.24166666667'
'England_CAMB_7291D_2020_United_Kingdom_B.1.1_2020.22777777778'
'England_CAMB_72D3F_2020_United_Kingdom_B.3_2020.225'
'England_CAMB_73026_2020_United_Kingdom_B.1_2020.22222222222'
'England_CAMB_731BA_2020_United_Kingdom_B.1_2020.225'
'England_CAMB_7368E_2020_United_Kingdom_B_2020.225'
'England_CAMB_739B2_2020_United_Kingdom_B.1.p11_2020.25'
'England_CAMB_73A0A_2020_United_Kingdom_B.2.1_2020.25'
'England_CAMB_73D3E_2020_United_Kingdom_B.1.1_2020.25'
'England_CAMB_73E4A_2020_United_Kingdom_B.1_2020.21666666667'
'England_CAMB_73F38_2020_United_Kingdom_B.2_2020.21944444444'
'England_CAMB_7417D_2020_United_Kingdom_B.2.6_2020.21944444444'
'England_CAMB_75992_2020_United_Kingdom_B.2.1_2020.25'
'England_CAMB_759FC_2020_United_Kingdom_B.1_2020.24444444444'
'England_CAMB_75B8D_2020_United_Kingdom_B.2.1_2020.24166666667'
'England_CAMB_75BC9_2020_United_Kingdom_B.3_2020.24444444444'
'England_CAMB_75D1E_2020_United_Kingdom_B.1.1_2020.24444444444'
'England_CAMB_75DD2_2020_United_Kingdom_B.1.1_2020.24444444444'
'England_CAMB_76454_2020_United_Kingdom_B.1_2020.24722222222'
'England_CAMB_76490_2020_United_Kingdom_B.1.1_2020.24722222222'
'England_CAMB_764FA_2020_United_Kingdom_B.1.1_2020.24444444444'
'England_CAMB_772FF_2020_United_Kingdom_B.1.5_2020.25'
'England_CAMB_77ABB_2020_United_Kingdom_B.1.1.7_2020.25'
'England_CAMB_77C79_2020_United_Kingdom_B.1.1_2020.25'
'England_CAMB_77DC1_2020_United_Kingdom_B.1_2020.25'
'England_CAMB_78337_2020_United_Kingdom_B.1.1.4_2020.25833333333'
'England_CAMB_78BA8_2020_United_Kingdom_B.1.1_2020.25277777778'
'England_CAMB_790D5_2020_United_Kingdom_B.1.1_2020.25555555556'
'England_CAMB_7949D_2020_United_Kingdom_B.1.1_2020.25555555556'
'England_CAMB_79873_2020_United_Kingdom_B.1.1.3_2020.25'
'England_CAMB_79BA7_2020_United_Kingdom_B.1.1.1_2020.25277777778'
'England_CAMB_79F14_2020_United_Kingdom_B.1_2020.25277777778'
'England_CAMB_79FF6_2020_United_Kingdom_B.1_2020.25277777778'
'England_CAMB_7A0F2_2020_United_Kingdom_B.2_2020.25277777778'
'England_CAMB_7A57B_2020_United_Kingdom_B.2.1_2020.25277777778'
'England_CAMB_7AAB8_2020_United_Kingdom_B.1.1_2020.26666666667'
'England_CAMB_7AD37_2020_United_Kingdom_B.1_2020.26944444444'
'England_CAMB_7B13A_2020_United_Kingdom_B.1.p11_2020.27777777778'
'England_CAMB_7B273_2020_United_Kingdom_B.1.1_2020.27777777778'

'England_CAMB_7B9F6_2020_United_Kingdom_B.1.p11_2020.25555555556'
'England_CAMB_7BF12_2020_United_Kingdom_B.1.1_2020.25833333333'
'England_CAMB_7C0D2_2020_United_Kingdom_B.1.1_2020.28611111111'
'England_CAMB_7CAD4_2020_United_Kingdom_B.1.1_2020.26111111111'
'England_CAMB_7D086_2020_United_Kingdom_B.1_2020.26111111111'
'England_CAMB_7D0FF_2020_United_Kingdom_B.1.1.1_2020.26388888889'
'England_CAMB_7D165_2020_United_Kingdom_B.1.1_2020.26388888889'
'England_CAMB_7D3C9_2020_United_Kingdom_B.1.1_2020.26388888889'
'England_CAMB_7D4F3_2020_United_Kingdom_B.1.1_2020.26388888889'
'England_CAMB_7D745_2020_United_Kingdom_B_2020.26388888889'
'England_CAMB_7D88E_2020_United_Kingdom_B.1.1.10_2020.26388888889'
'England_CAMB_7DC19_2020_United_Kingdom_B.1.1_2020.26388888889'
'England_CAMB_7EB48_2020_United_Kingdom_B.1.1_2020.26666666667'
'England_CAMB_7F215_2020_United_Kingdom_B.1.1_2020.26944444444'
'England_CAMB_7F497_2020_United_Kingdom_B.1_2020.26944444444'
'England_CAMB_81122_2020_United_Kingdom_B.1.1_2020.30277777778'
'England_CAMB_81298_2020_United_Kingdom_B.1.1_2020.275'
'England_CAMB_81896_2020_United_Kingdom_B.2_2020.28055555556'
'England_CAMB_818E1_2020_United_Kingdom_B.1.1.13_2020.28055555556'
'England_CAMB_8191B_2020_United_Kingdom_B.2.1_2020.28055555556'
'England_CAMB_81BD9_2020_United_Kingdom_B.1.1_2020.27777777778'
'England_CAMB_81E49_2020_United_Kingdom_B.1.1_2020.30833333333'
'England_CAMB_81FDD_2020_United_Kingdom_B.1.p11_2020.30833333333'
'England_CAMB_823A3_2020_United_Kingdom_B.1_2020.28333333333'
'England_CAMB_8266E_2020_United_Kingdom_B.1_2020.28333333333'
'England_CAMB_82E39_2020_United_Kingdom_B.1.1_2020.28611111111'
'England_CAMB_831E4_2020_United_Kingdom_B.1.1_2020.28611111111'
'England_CAMB_83436_2020_United_Kingdom_B.1_2020.28611111111'
'England_CAMB_837A6_2020_United_Kingdom_B.1.1_2020.28888888889'
'England_CAMB_83849_2020_United_Kingdom_B.1.1.13_2020.28333333333'
'England_CAMB_839EC_2020_United_Kingdom_B.1.1_2020.29166666667'
'England_CAMB_843A1_2020_United_Kingdom_B.2.1_2020.29166666667'
'England_CAMB_84DB2_2020_United_Kingdom_B.2_2020.19722222222'
'England_CAMB_84E55_2020_United_Kingdom_B.1.1_2020.19444444444'
'England_EXET_13562D_2020_United_Kingdom_B.1.1_2020.26388888889'
'England_EXET_1356E1_2020_United_Kingdom_B.1.1_2020.28055555556'
'England_EXET_1358BE_2020_United_Kingdom_B.1_2020.28888888889'
'England_EXET_135B88_2020_United_Kingdom_B.1_2020.25'
'England_EXET_135C3A_2020_United_Kingdom_B.1.1_2020.275'
'England_EXET_136AE4_2020_United_Kingdom_B.1.1_2020.31111111111'
'England_LIVE_99479_2020_United_Kingdom_B.1.1_2020.26944444444'
'England_LIVE_99594_2020_United_Kingdom_B_2020.26944444444'
'England_LIVE_99691_2020_United_Kingdom_B.1.1_2020.27222222222'
'England_LIVE_99743_2020_United_Kingdom_B.1.1_2020.27222222222'
'England_LIVE_997DA_2020_United_Kingdom_B.1.1.1_2020.27222222222'
'England_LIVE_99A3B_2020_United_Kingdom_B.1.1_2020.28055555556'
'England_LIVE_99D6F_2020_United_Kingdom_B.1.1.1_2020.28333333333'
'England_LIVE_9A0ED_2020_United_Kingdom_B.1.1_2020.29444444444'

'England_LIVE_9A302_2020_United_Kingdom_B.1_2020.29166666667'
'England_LIVE_9A311_2020_United_Kingdom_B.1_2020.28888888889'
'England_LIVE_9AC52_2020_United_Kingdom_B.1.1_2020.25'
'England_LIVE_9AC70_2020_United_Kingdom_B.1.1_2020.24722222222'
'England_LIVE_9B037_2020_United_Kingdom_B.1.1_2020.24166666667'
'England_LIVE_9B107_2020_United_Kingdom_B.1.1.1_2020.23333333333'
'England_LIVE_9B389_2020_United_Kingdom_B.1.1_2020.23888888889'
'England_LIVE_9B3E3_2020_United_Kingdom_B.1.1_2020.25555555556'
'England_LIVE_9B44A_2020_United_Kingdom_B.1_2020.25277777778'
'England_LIVE_9B583_2020_United_Kingdom_B.1.1_2020.25277777778'
'England_LIVE_9B680_2020_United_Kingdom_B.1.1_2020.3'
'England_LIVE_9B69F_2020_United_Kingdom_B.1_2020.30277777778'
'England_LIVE_9BD9A_2020_United_Kingdom_B.1_2020.30277777778'
'England_LIVE_9C3E2_2020_United_Kingdom_B_2020.24444444444'
'England_LIVE_9C555_2020_United_Kingdom_B.1_2020.22777777778'
'England_LIVE_9C889_2020_United_Kingdom_B.1.1.1_2020.22777777778'
'England_LIVE_9D40C_2020_United_Kingdom_B.1.1_2020.28888888889'
'England_LIVE_9E070_2020_United_Kingdom_B.1.1.1_2020.25277777778'
'England_LIVE_9E16E_2020_United_Kingdom_B.1_2020.25555555556'
'England_LIVE_9E580_2020_United_Kingdom_B.1.1_2020.25833333333'
'England_LIVE_9E5F9_2020_United_Kingdom_B.1_2020.27777777778'
'England_LIVE_9EDB5_2020_United_Kingdom_B.1_2020.25833333333'
'England_LIVE_9EFCE_2020_United_Kingdom_B.1.1.1_2020.275'
'England_LIVE_9F0F7_2020_United_Kingdom_B_2020.34444444444'
'England_LOND_D358F_2020_United_Kingdom_B.1_2020.23888888889'
'England_LOND_D3D87_2020_United_Kingdom_B.2.5_2020.25833333333'
'England_LOND_D406F_2020_United_Kingdom_B.1_2020.25833333333'
'England_LOND_D5909_2020_United_Kingdom_B.1.1.1_2020.29444444444'
'England_LOND_D59AF_2020_United_Kingdom_B.1.1_2020.29444444444'
'England_LOND_D6030_2020_United_Kingdom_B.1.1_2020.30277777778'
'England_LOND_D604F_2020_United_Kingdom_A_2020.3'
'England_LOND_D6AC9_2020_United_Kingdom_B.1.1_2020.36944444444'
'England_NORT_288D52_2020_United_Kingdom_B.2_2020.22222222222'
'England_NORT_28ABDE_2020_United_Kingdom_B.1_2020.28888888889'
'England_NORT_292317_2020_United_Kingdom_B.1.1_2020.35555555556'
'England_NORW_E6E33_2020_United_Kingdom_B.1_2020.30555555556'
'England_NORW_E70E1_2020_United_Kingdom_B.1.1_2020.3'
'England_NORW_E738E_2020_United_Kingdom_B.1.1_2020.30277777778'
'England_NORW_E73F7_2020_United_Kingdom_B.1.5.5_2020.33888888889'
'England_NORW_E7430_2020_United_Kingdom_B.1.1_2020.33888888889'
'England_NORW_E75E2_2020_United_Kingdom_B.1.5.5_2020.34166666667'
'England_NORW_E7737_2020_United_Kingdom_B.1.1_2020.34166666667'
'England_NORW_E7755_2020_United_Kingdom_B.1_2020.34166666667'
'England_NORW_E7922_2020_United_Kingdom_B.1_2020.34444444444'
'England_NORW_E797D_2020_United_Kingdom_B.1.1_2020.34444444444'
'England_NORW_E85B4_2020_United_Kingdom_B.2_2020.29722222222'
'England_NORW_E888E_2020_United_Kingdom_B.1.1_2020.28055555556'
'England_NORW_E89C7_2020_United_Kingdom_B.1.1_2020.27777777778'

'England_NORW_E8B94_2020_United_Kingdom_B.2_2020.28055555556'
'England_NORW_E8CBF_2020_United_Kingdom_B_2020.28333333333'
'England_NORW_E8DBC_2020_United_Kingdom_B.1.1_2020.32222222222'
'England_NORW_E94B6_2020_United_Kingdom_B.2_2020.30833333333'
'England_NORW_EAD23_2020_United_Kingdom_B.2_2020.32777777778'
'England_NORW_EBBBF_2020_United_Kingdom_B.1.1_2020.34722222222'
'England_NORW_EC732_2020_United_Kingdom_B.1.1_2020.36388888889'
'England_NOTT_10E11D_2020_United_Kingdom_B_2020.20277777778'
'England_NOTT_10E14A_2020_United_Kingdom_B.2.5_2020.21111111111'
'England_NOTT_10E283_2020_United_Kingdom_B.1_2020.21944444444'
'England_NOTT_10E2A1_2020_United_Kingdom_B.2.2_2020.22222222222'
'England_NOTT_10F6FF_2020_United_Kingdom_B.1.34_2020.25833333333'
'England_NOTT_10FB96_2020_United_Kingdom_B_2020.26666666667'
'England_NOTT_1103B8_2020_United_Kingdom_B.1_2020.29166666667'
'England_NOTT_110D50_2020_United_Kingdom_B_2020.32777777778'
'England_NOTT_110E3F_2020_United_Kingdom_B_2020.32777777778'
'England_NOTT_11127E_2020_United_Kingdom_B.1.1_2020.31111111111'
'England_NOTT_11138A_2020_United_Kingdom_B.1_2020.36666666667'
'England_NOTT_1115DF_2020_United_Kingdom_B.1.1.9_2020.38055555556'
'England_NOTT_1116CD_2020_United_Kingdom_B.1.1_2020.38888888889'
'England_NOTT_1116EB_2020_United_Kingdom_B.1.1_2020.39166666667'
'England_NOTT_111AC1_2020_United_Kingdom_B.1.1_2020.40555555556'
'England_OXON_AC622_2020_United_Kingdom_B.1.1_2020.26666666667'
'England_OXON_ACADB_2020_United_Kingdom_B.1.1_2020.26111111111'
'England_OXON_ACB9C_2020_United_Kingdom_B.1_2020.25833333333'
'England_OXON_AEC79_2020_United_Kingdom_B.1.1.9_2020.26944444444'
'England_PORT_2CF4C3_2020_United_Kingdom_B.1.1_2020.32222222222'
'England_SHEF_BFE06_2020_United_Kingdom_B.2.1_2020.21111111111'
'England_SHEF_C0303_2020_United_Kingdom_B.1_2020.23888888889'
'England_SHEF_C15FD_2020_United_Kingdom_B.1_2020.24722222222'
'England_SHEF_C18F4_2020_United_Kingdom_B.1_2020.27777777778'
'England_SHEF_C1F3B_2020_United_Kingdom_B.3_2020.23055555556'
'England_SHEF_C2D03_2020_United_Kingdom_B.1.1_2020.25'
'England_SHEF_C30DC_2020_United_Kingdom_B.1.1_2020.225'
'England_SHEF_C41BA_2020_United_Kingdom_B.1.1_2020.27222222222'
'England_SHEF_C42C6_2020_United_Kingdom_B.1_2020.28611111111'
'England_SHEF_C4396_2020_United_Kingdom_B.1.1_2020.25'
'England_SHEF_C4C7D_2020_United_Kingdom_B_2020.30277777778'
'England_SHEF_C5070_2020_United_Kingdom_B.1.1_2020.28055555556'
'England_SHEF_C5D6A_2020_United_Kingdom_B.1.1.1_2020.23055555556'
'England_SHEF_C6C02_2020_United_Kingdom_B.1.1.1_2020.27777777778'
'England_SHEF_C774C_2020_United_Kingdom_B.2.1_2020.21666666667'
'England_SHEF_C88A2_2020_United_Kingdom_B.1.1.5_2020.30555555556'
'England_SHEF_C906D_2020_United_Kingdom_B.1.1_2020.28333333333'
'England_SHEF_C9F33_2020_United_Kingdom_B.1.1.1_2020.25277777778'
'England_SHEF_CB1A4_2020_United_Kingdom_B.1_2020.26111111111'
'England_SHEF_CB423_2020_United_Kingdom_B.1.1_2020.30833333333'
'England_SHEF_CD39D_2020_United_Kingdom_B.2.2_2020.24444444444'

'England_SHEF_CDD17_2020_United_Kingdom_B.1.1.5_2020.30555555556'
'England_SHEF_CDEE7_2020_United_Kingdom_B.1.1_2020.275'
'England_SHEF_CE684_2020_United_Kingdom_B.2.1_2020.25833333333'
'England_SHEF_D1A55_2020_United_Kingdom_B.1.1.1_2020.26388888889'
'England_SHEF_D2BF7_2020_United_Kingdom_B.1.1_2020.26944444444'
'Finland_14M74_2020_Finland_B.3_2020.20277777778'
'France_ARA12759_2020_France_B.1_2020.22777777778'
'France_B5355_2020_France_B.1.1_2020.23055555556'
'France_HF3295_2020_France_B.1_2020.22777777778'
'Germany_BAV_MVP0001_2020_Germany_B.2.1_2020.20277777778'
'Germany_BAV_MVP0045_2020_Germany_B.1.1_2020.18611111111'
'Germany_BAV_MVP0052_2020_Germany_B.1.1_2020.20277777778'
'Germany_BAV_MVP0059_2020_Germany_B.1_2020.21944444444'
'Germany_BAV_MVP0240_2020_Germany_B.1_2020.325'
'Germany_NRW_55_2020_Germany_B.1_2020.21944444444'
'Ghana_1651_S3_2020_Ghana_A_2020.23333333333'
'Ghana_2828_S6_2020_Ghana_A_2020.24444444444'
'Ghana_2850_S15_2020_Ghana_A_2020.24444444444'
'Ghana_2944_S9_2020_Ghana_B.1_2020.24722222222'
'Ghana_2986_S10_2020_Ghana_B.1_2020.25'
'Ghana_3177_S12_2020_Ghana_A_2020.24722222222'
'Greece_208_33922_2020_Greece_B.1.1_2020.20277777778'
'Greece_38_2020_Greece_B.1.5_2020.22777777778'
'Guangdong_2020XN4475_P0042_2020_China_A_2020.08055555556'
'Guangdong_20SF758_2020_China_B_2020.07222222222'
'Guangdong_SZ_N59_P0049_2020_China_B_2020.15833333333'
'Guangzhou_20SF206_2020_China_B_2020.05833333333'
'Guangzhou_GZMU0036_2020_China_B_2020.08333333333'
'Guangzhou_GZMU0077_2020_China_A_2020.11111111111'
'Hangzhou_HZCDC0090L_2020_China_B_2020.05555555556'
'HongKong_HKU_001a_2020_Hong_Kong_A_2020.05833333333'
'HongKong_VM20009579_2020_Hong_Kong_B.1.1_2020.20833333333'
'ITALY_SardiniaNuoro04039046_2020_Italy_B.1.1_2020.25555555556'
'Iceland_155_2020_Iceland_B.1_2020.20555555556'
'Iceland_163_2020_Iceland_B.1_2020.20277777778'
'Iceland_197_2020_Iceland_B.1_2020.20833333333'
'Iceland_232_2020_Iceland_B.1_2020.21111111111'
'Iceland_233_2020_Iceland_B.1.1_2020.21111111111'
'Iceland_247_2020_Iceland_B.1.1_2020.21111111111'
'Iceland_286_2020_Iceland_B.1_2020.21388888889'
'Iceland_423_2020_Iceland_B.1.5.1_2020.21666666667'
'Iceland_493_2020_Iceland_B.1.5.1_2020.21944444444'
'Iceland_517_2020_Iceland_B_2020.23333333333'
'Iceland_55_2020_Iceland_B.1_2020.18055555556'
'Iceland_97_2020_Iceland_B.1.1.17_2020.19166666667'
'Iceland_9_2020_Iceland_A.1.1_2020.19444444444'
'India_1617_2020_India_B.4_2020.19722222222'
'India_1_27_2020_India_B_2020.07222222222'

'India_CCMB_L988_2020_India_B.1_2020.36111111111'
'India_CCMB_NIV5_2020_India_B.1_2020.21111111111'
'India_CCMB_O1_2020_India_B.6_2020.28333333333'
'India_GBRC129_2020_India_B.1_2020.33611111111'
'India_GBRC165_2020_India_B.1.36_2020.40555555556'
'India_GBRC166b_2020_India_B.1.36_2020.40555555556'
'India_GBRC171_2020_India_B.1.36_2020.42777777778'
'India_GBRC29_2020_India_B.1_2020.33055555556'
'India_GBRC2_2020_India_B.1_2020.28333333333'
'India_GBRC47_2020_India_B.1_2020.33055555556'
'India_GBRC74_2020_India_B.1_2020.33611111111'
'India_ILSCV13921_2020_India_B.6_2020.35833333333'
'India_NCDC_02415_2020_India_A.2_2020.24722222222'
'India_NCDC_3175_2020_India_B.6_2020.26111111111'
'India_NGC_CDFD_32_2020_India_B.1.1.8_2020.41388888889'
'India_NIV_6614_2020_India_B.6_2020.25277777778'
'India_S16_2020_India_B.1_2020.25555555556'
'India_S58_2020_India_B.1_2020.33888888889'
'India_THSTI_BAL_42_2020_India_B.6_2020.26666666667'
'India_nimh_0318_2020_India_B.6_2020.275'
'Israel_13077560_2020_Israel_B.1_2020.25555555556'
'Israel_2020069_2020_Israel_B.6_2020.21111111111'
'Israel_2020084_2020_Israel_B.1_2020.21111111111'
'Israel_2086004_2020_Israel_B.1.3_2020.25'
'Israel_2086034_2020_Israel_B.1_2020.25'
'Israel_2089723_2020_Israel_B.1.3_2020.25'
'Israel_701002550_2020_Israel_B.1_2020.23611111111'
'Italy_484_2020_Italy_B.1_2020.15555555556'
'Jamaica_JM_CDC_4376_2020_Jamaica_B.6_2020.19444444444'
'Japan_DP0027_2020_Japan_B_2020.12222222222'
'Japan_DP0190_2020_Japan_B_2020.12222222222'
'Japan_Donner3_2020_Japan_B.1.1_2020.26388888889'
'Jiangsu_JS02_2020_China_B_2020.06388888889'
'Kazakhstan_26530_2020_Kazakhstan_B.4_2020.31944444444'
'Latvia_01_2020_Latvia_B.1_2020.23333333333'
'Latvia_04_2020_Latvia_B.3_2020.23333333333'
'Luxembourg_LNS0001029_2020_Luxembourg_B.1.1_2020.325'
'Luxembourg_LNS0516520_2020_Luxembourg_B.1_2020.25'
'Luxembourg_LNS0877496_2020_Luxembourg_B.1_2020.21111111111'
'Luxembourg_LNS2458393_2020_Luxembourg_B.1_2020.28611111111'
'Luxembourg_LNS2913521_2020_Luxembourg_B.1_2020.23611111111'
'Luxembourg_LNS6854244_2020_Luxembourg_B.1_2020.25'
'Luxembourg_LNS6944114_2020_Luxembourg_B.1_2020.24722222222'
'Luxembourg_LNS8705424_2020_Luxembourg_B.1_2020.31111111111'
'Luxembourg_LNS9831617_2020_Luxembourg_B.1_2020.26944444444'
'Netherlands_Berlicum_1363564_2020_Netherlands_B.1.1_2020.14722222222'
'Netherlands_Gelderland_81_2020_Netherlands_B.1.22_2020.38333333333'
'Netherlands_Gelderland_95_2020_Netherlands_B.1.22_2020.38888888889'

'Netherlands_Gelderland_96_2020_Netherlands_B.1.22_2020.38888888889'
'Netherlands_Limburg_10_2020_Netherlands_B.1.8_2020.21944444444'
'Netherlands_Limburg_26_2020_Netherlands_B.1_2020.3'
'Netherlands_NA_176_2020_Netherlands_B.1_2020.225'
'Netherlands_NA_177_2020_Netherlands_B.1_2020.225'
'Netherlands_NA_187_2020_Netherlands_B.1.8_2020.22777777778'
'Netherlands_NA_199_2020_Netherlands_B.2_2020.23333333333'
'Netherlands_NA_235_2020_Netherlands_B.1.p9_2020.23888888889'
'Netherlands_NA_333_2020_Netherlands_B_2020.25555555556'
'Netherlands_NA_375_2020_Netherlands_B.1.1_2020.26944444444'
'Netherlands_NA_501_2020_Netherlands_B.1_2020.23055555556'
'Netherlands_NA_512_2020_Netherlands_B.1_2020.25'
'Netherlands_NA_632_2020_Netherlands_B.1.1_2020.25833333333'
'Netherlands_NA_72_2020_Netherlands_B.2_2020.19166666667'
'Netherlands_NA_77_2020_Netherlands_B_2020.18888888889'
'Netherlands_NA_82_2020_Netherlands_B_2020.19444444444'
'Netherlands_NA_83_2020_Netherlands_B_2020.19444444444'
'Netherlands_NoordBrabant_103_2020_Netherlands_B.1.8_2020.18888888889'
'Netherlands_NoordHolland_20_2020_Netherlands_B.1.22_2020.31388888889'
'Netherlands_Overijssel_5_2020_Netherlands_B.1_2020.26111111111'
'Netherlands_Tilburg_1363354_2020_Netherlands_B_2020.15555555556'
'Netherlands_Utrecht_10016_2020_Netherlands_B.1.1_2020.21388888889'
'Netherlands_Utrecht_10028_2020_Netherlands_B.1.1_2020.25'
'Netherlands_Utrecht_1363564_2020_Netherlands_B.1_2020.16666666667'
'Netherlands_Zeeland_33_2020_Netherlands_B.1_2020.31666666667'
'Netherlands_Zeeland_47_2020_Netherlands_B.1_2020.24722222222'
'Netherlands_ZuidHolland_146_2020_Netherlands_B.1_2020.3'
'Netherlands_ZuidHolland_152_2020_Netherlands_B.1.1.10_2020.325'
'Netherlands_ZuidHolland_218_2020_Netherlands_B.1.22_2020.35555555556'
'Netherlands_ZuidHolland_63_2020_Netherlands_B_2020.22777777778'
'Netherlands_ZuidHolland_89_2020_Netherlands_B.1.22_2020.23888888889'
'Netherlands_ZuidHolland_90_2020_Netherlands_B.1.5.2_2020.24444444444'
'Netherlands_ZuidHolland_94_2020_Netherlands_B_2020.25'
'NorthernIreland_NIRE_101E7D_2020_United_Kingdom_B.1.1.10_2020.23055555556'
'NorthernIreland_NIRE_10235F_2020_United_Kingdom_B.1.1.1_2020.225'
'NorthernIreland_NIRE_FA69E_2020_United_Kingdom_B.1.1.1_2020.25'
'NorthernIreland_NIRE_FA7D7_2020_United_Kingdom_B.1.1_2020.24722222222'
'NorthernIreland_NIRE_FAC8D_2020_United_Kingdom_B.10_2020.23611111111'
'NorthernIreland_NIRE_FB4EE_2020_United_Kingdom_B.10_2020.20833333333'
'NorthernIreland_NIRE_FC22F_2020_United_Kingdom_B.1_2020.27777777778'
'NorthernIreland_NIRE_FC429_2020_United_Kingdom_B.1.1_2020.28055555556'
'NorthernIreland_NIRE_FC438_2020_United_Kingdom_B.1.1_2020.28055555556'
'Norway_2386_2020_Norway_B.1_2020.25833333333'
'Oman_205034603_2020_Oman_B.1.1_2020.35555555556'
'Oman_RESP_20_6417_2020_Oman_B.1_2020.24444444444'
'Philippines_PGC04_2020_Philippines_B.6_2020.23611111111'
'Philippines_RITM_02_2020_Philippines_B.6_2020.19444444444'
'Poland_Sla8_2020_Poland_B.1.1_2020.34444444444'

'Portugal_PT0174_2020_Portugal_B.2.1_2020.24166666667'
'Portugal_PT0184_2020_Portugal_B.1_2020.24166666667'
'Portugal_PT0187_2020_Portugal_B.1.1_2020.24444444444'
'Portugal_PT0225_2020_Portugal_B.1.1_2020.25'
'Portugal_PT0266_2020_Portugal_B.1_2020.25833333333'
'Portugal_PT0326_2020_Portugal_B.1.1_2020.20833333333'
'Portugal_PT0446_2020_Portugal_B.1.1_2020.23888888889'
'Portugal_PT0613_2020_Portugal_B.1_2020.20555555556'
'Portugal_PT0616_2020_Portugal_B.1.1_2020.21111111111'
'Qatar_QA18_2020_Qatar_B_2020.22777777778'
'Russia_CRIE139642_2020_Russia_B.1.1_2020.24444444444'
'Russia_SCPM_O_02_2020_Russia_B.1_2020.225'
'Russia_StPetersburg_RII4328S_2020_Russia_B.1.1_2020.23333333333'
'Russia_StPetersburg_RII4969S_2020_Russia_B.1.1_2020.275'
'SaudiArabia_KAUST_Makkah05_2020_Saudi_Arabia_A_2020.22777777778'
'SaudiArabia_KAUST_Makkah204_2020_Saudi_Arabia_B.1.36_2020.28611111111'
'Scotland_CVR1115_2020_United_Kingdom_B.16_2020.24444444444'
'Scotland_CVR1138_2020_United_Kingdom_B.2_2020.24722222222'
'Scotland_CVR1345_2020_United_Kingdom_B.1_2020.25'
'Scotland_CVR1408_2020_United_Kingdom_B.1.p73_2020.25'
'Scotland_CVR1499_2020_United_Kingdom_A.3_2020.25555555556'
'Scotland_CVR1536_2020_United_Kingdom_B.1_2020.25833333333'
'Scotland_CVR1587_2020_United_Kingdom_B.2_2020.26111111111'
'Scotland_CVR1647_2020_United_Kingdom_B.3_2020.26388888889'
'Scotland_CVR21_2020_United_Kingdom_B.1.1_2020.2'
'Scotland_CVR2282_2020_United_Kingdom_B.1.p73_2020.28055555556'
'Scotland_CVR2314_2020_United_Kingdom_B.1.36_2020.28333333333'
'Scotland_CVR235_2020_United_Kingdom_B.1_2020.21666666667'
'Scotland_CVR2375_2020_United_Kingdom_A.2_2020.28333333333'
'Scotland_CVR2415_2020_United_Kingdom_B.16_2020.28611111111'
'Scotland_CVR2510_2020_United_Kingdom_A.3_2020.28888888889'
'Scotland_CVR2740_2020_United_Kingdom_B.1_2020.29722222222'
'Scotland_CVR2764_2020_United_Kingdom_B.1.1.1_2020.30277777778'
'Scotland_CVR2973_2020_United_Kingdom_B.1.1.14_2020.30833333333'
'Scotland_CVR3015_2020_United_Kingdom_B.1_2020.31111111111'
'Scotland_CVR3084_2020_United_Kingdom_B.1.1_2020.31388888889'
'Scotland_CVR3127_2020_United_Kingdom_B.1.p73_2020.31944444444'
'Scotland_CVR3191_2020_United_Kingdom_B.1_2020.325'
'Scotland_CVR3209_2020_United_Kingdom_B.1.p73_2020.29166666667'
'Scotland_CVR3411_2020_United_Kingdom_B.16_2020.35'
'Scotland_CVR3482_2020_United_Kingdom_B.1_2020.36388888889'
'Scotland_CVR3492_2020_United_Kingdom_B.1.1.1_2020.3'
'Scotland_CVR465_2020_United_Kingdom_B.2.1_2020.22777777778'
'Scotland_CVR62_2020_United_Kingdom_B.1_2020.19722222222'
'Scotland_CVR712_2020_United_Kingdom_B.16_2020.23611111111'
'Scotland_EDB006_2020_United_Kingdom_B.1.1_2020.18888888889'
'Scotland_EDB008_2020_United_Kingdom_B.1.1_2020.18888888889'
'Scotland_EDB012_2020_United_Kingdom_B_2020.18333333333'

'Scotland_EDB028_2020_United_Kingdom_B.1.1_2020.19722222222'
'Scotland_EDB048_2020_United_Kingdom_B.2_2020.21111111111'
'Scotland_EDB049_2020_United_Kingdom_B.1_2020.20555555556'
'Scotland_EDB1078_2020_United_Kingdom_B.1.5.6_2020.26666666667'
'Scotland_EDB1830_2020_United_Kingdom_B.1.5_2020.29166666667'
'Scotland_EDB2011_2020_United_Kingdom_B.1.5_2020.29722222222'
'Scotland_EDB2445_2020_United_Kingdom_B.1_2020.30833333333'
'Scotland_EDB254_2020_United_Kingdom_B.1.p11_2020.23611111111'
'Scotland_EDB3558_2020_United_Kingdom_B.1.p11_2020.28611111111'
'Scotland_EDB3662_2020_United_Kingdom_B.1.5_2020.325'
'Scotland_EDB3740_2020_United_Kingdom_B_2020.325'
'Scotland_EDB3778_2020_United_Kingdom_B.1_2020.32777777778'
'Scotland_EDB3899_2020_United_Kingdom_B_2020.33333333333'
'Scotland_EDB401_2020_United_Kingdom_B_2020.24444444444'
'Scotland_EDB4357_2020_United_Kingdom_B.1.5_2020.31388888889'
'Scotland_EDB4551_2020_United_Kingdom_B.1_2020.34722222222'
'Scotland_EDB5181_2020_United_Kingdom_B.1.5_2020.38333333333'
'Scotland_EDB5245_2020_United_Kingdom_B.1.5_2020.38611111111'
'Scotland_EDB5466_2020_United_Kingdom_B.1.1.1_2020.31666666667'
'Scotland_EDB805_2020_United_Kingdom_B.1_2020.25555555556'
'Scotland_GCVR_1700A0_2020_United_Kingdom_B.1_2020.23055555556'
'Scotland_GCVR_170617_2020_United_Kingdom_B.1.1.1_2020.23055555556'
'Scotland_GCVR_170BEB_2020_United_Kingdom_B.16_2020.23333333333'
'Scotland_GCVR_1712A9_2020_United_Kingdom_B_2020.23611111111'
'Scotland_GCVR_17132E_2020_United_Kingdom_B.1.5_2020.23611111111'
'Scotland_GCVR_173BCA_2020_United_Kingdom_B.1_2020.26388888889'
'Senegal_315_2020_Senegal_B.1_2020.21111111111'
'Senegal_620_2020_Senegal_A_2020.21944444444'
'Shanghai_SH0050_2020_China_B_2020.09444444444'
'Shanghai_SH0075_2020_China_A_2020.08055555556'
'Shanghai_SH0119_2020_China_B_2020.10555555556'
'Shanghai_SH0126_2020_China_B.4_2020.12222222222'
'Shaoxing_06_2020_China_A_2020.08333333333'
'Singapore_128_2020_Singapore_B.1.1_2020.22222222222'
'Singapore_142_2020_Singapore_B.1.1_2020.21111111111'
'Singapore_159_2020_Singapore_B_2020.21666666667'
'Singapore_273_2020_Singapore_B.1.1.1_2020.22222222222'
'Singapore_312_2020_Singapore_B.6_2020.28611111111'
'Singapore_326_2020_Singapore_B.6_2020.29444444444'
'Singapore_55_2020_Singapore_B.6_2020.26944444444'
'Singapore_93_2020_Singapore_B.2.1_2020.21388888889'
'SouthAfrica_KRISP_0153_2020_South_Africa_B.1.1_2020.41666666667'
'SouthAfrica_KRISP_0158_2020_South_Africa_B.1_2020.40833333333'
'SouthAfrica_Tygerberg_01_2020_South_Africa_B.1.8_2020.21111111111'
'Spain_Alcaniz2479_2020_Spain_B.1.5_2020.23888888889'
'Spain_Barcelona_VH9461_2020_Spain_B.1.5_2020.27222222222'
'Spain_COV000706_2020_Spain_A.2_2020.17777777778'
'Spain_COV000784_2020_Spain_A.2_2020.18888888889'

'Spain_COV000926_2020_Spain_B.1.1_2020.23888888889'
'Spain_COV000944_2020_Spain_A.2_2020.24166666667'
'Spain_COV001164_2020_Spain_B.1_2020.23333333333'
'Spain_COV001323_2020_Spain_B.1.5_2020.22777777778'
'Spain_COV001368_2020_Spain_B.1.5_2020.19722222222'
'Spain_COV001893_2020_Spain_A.2_2020.2'
'Spain_COV001932_2020_Spain_A.2_2020.19444444444'
'Spain_COV003650_2020_Spain_B.1.5_2020.25'
'Spain_Donostia_San_Sebastian2077_2020_Spain_B.1.5_2020.275'
'Spain_Donostia_San_Sebastian2086_2020_Spain_B.1.1_2020.275'
'Spain_Donostia_San_Sebastian2114_2020_Spain_B.1.1_2020.27777777778'
'Spain_Donostia_San_Sebastian2152_2020_Spain_B.1.1_2020.28055555556'
'Spain_Galicia202076_2020_Spain_B.1.5_2020.19166666667'
'Spain_Ibiza2503_2020_Spain_A.2_2020.21666666667'
'Spain_LaRioja201568_2020_Spain_A_2020.17777777778'
'Spain_Valencia155_2020_Spain_B.1.5_2020.21666666667'
'Spain_Valencia171_2020_Spain_B.1.1_2020.21944444444'
'Spain_Valencia189_2020_Spain_A.5_2020.23611111111'
'Spain_Valencia213_2020_Spain_B.1_2020.24166666667'
'Spain_Valencia283_2020_Spain_B.1_2020.21111111111'
'Spain_Valencia291_2020_Spain_B.1_2020.21111111111'
'Spain_Valencia304_2020_Spain_A.5_2020.225'
'Spain_Valencia309_2020_Spain_A.5_2020.225'
'Spain_Valencia40_2020_Spain_B.1_2020.18333333333'
'Spain_Valencia81_2020_Spain_B.1.1_2020.21944444444'
'Spain_Valencia83_2020_Spain_B.1.5_2020.22222222222'
'Spain_Valencia94_2020_Spain_A.5_2020.21111111111'
'Spain_Zaragoza2416_2020_Spain_B_2020.22222222222'
'Spain_Zaragoza2447_2020_Spain_B_2020.21388888889'
'Sweden_20_08100_2020_Sweden_B.1.1_2020.18055555556'
'Sweden_20_08156_2020_Sweden_B.1_2020.19444444444'
'Sweden_20_50094_2020_Sweden_B.1.1_2020.175'
'Sweden_20_50223_2020_Sweden_B.1_2020.18611111111'
'Sweden_20_50268_2020_Sweden_B.1.1_2020.18333333333'
'Sweden_20_50315_2020_Sweden_B.1_2020.19722222222'
'Switzerland_100026_2020_Switzerland_B.1_2020.18888888889'
'Switzerland_100091_2020_Switzerland_B.1_2020.2'
'Switzerland_100819_2020_Switzerland_B.1_2020.23611111111'
'Switzerland_100843_2020_Switzerland_B.1_2020.23333333333'
'Switzerland_110448_2020_Switzerland_B.1_2020.26666666667'
'Switzerland_120047_2020_Switzerland_B.1.1_2020.30277777778'
'Switzerland_GE2297_2020_Switzerland_B.1.12_2020.24444444444'
'Switzerland_GE4644_2020_Switzerland_B.1.1_2020.26666666667'
'Switzerland_GR2988_2020_Switzerland_B.1.1_2020.15555555556'
'Taiwan_108_2020_Taiwan_B.1_2020.22777777778'
'Taiwan_299_2020_Taiwan_B.6_2020.24444444444'
'Taiwan_CGMH_CGU_18_2020_Taiwan_B.1.1_2020.21388888889'
'Taiwan_TSGH_03_2020_Taiwan_B.1.1_2020.21111111111'

'Thailand_Bangkok_0060_2020_Thailand_A.6_2020.24166666667'
'Thailand_Bangkok_0065_2020_Thailand_A.6_2020.24444444444'
'Thailand_Bangkok_580_2020_Thailand_A_2020.08333333333'
'Thailand_Bangkok_CONI_0142_2020_Thailand_A.6_2020.20277777778'
'Thailand_NIH_2420_2020_Thailand_A.6_2020.2'
'Turkey_HSGM_1014_2020_Turkey_B.1.5_2020.23333333333'
'Turkey_HSGM_5770_2020_Turkey_A_2020.21666666667'
'USA_AK_PHL180_2020_USA_B.1_2020.26944444444'
'USA_CA_CZB07_2020_USA_B.1.1_2020.21388888889'
'USA_CA_CZB_1051_2020_USA_B_2020.26388888889'
'USA_CA_CZB_1080_2020_USA_B_2020.25833333333'
'USA_CA_CZB_1088_2020_USA_B_2020.26111111111'
'USA_CA_CZB_1092_2020_USA_B_2020.33333333333'
'USA_CA_CZB_1128_2020_USA_B.1_2020.26111111111'
'USA_CA_CZB_1208_2020_USA_A.1_2020.22777777778'
'USA_CA_CZB_1216_2020_USA_B.1_2020.23055555556'
'USA_CA_CZB_1233_2020_USA_B.14_2020.275'
'USA_CA_CZB_1278_2020_USA_A.1_2020.23888888889'
'USA_CA_CZB_1479_2020_USA_B.1_2020.33333333333'
'USA_CA_CZB_1506_2020_USA_B.1_2020.25'
'USA_CA_CZB_1513_2020_USA_A.3_2020.28333333333'
'USA_CA_CZB_1591_2020_USA_B_2020.23611111111'
'USA_CA_CZB_1607_2020_USA_A.1_2020.26388888889'
'USA_CA_CZB_1621_2020_USA_B.1.3_2020.33333333333'
'USA_CA_CZB_1657_2020_USA_B.1_2020.30833333333'
'USA_CA_CZB_1677_2020_USA_B.1_2020.24722222222'
'USA_CA_QDX_148_2020_USA_A.3_2020.20833333333'
'USA_CA_QDX_51_2020_USA_B.6_2020.20555555556'
'USA_CA_SCCPHD_UC140_2020_USA_A.1_2020.19166666667'
'USA_CA_SR0128_2020_USA_B.1_2020.24166666667'
'USA_CT_UW260_2020_USA_A.3_2020.20277777778'
'USA_CT_UW_4346_2020_USA_B.1.26_2020.24722222222'
'USA_CT_UW_4347_2020_USA_B.1_2020.24722222222'
'USA_CT_UW_6380_2020_USA_B.1_2020.25'
'USA_CT_UW_6568_2020_USA_B.1_2020.25277777778'
'USA_CT_UW_6590_2020_USA_B.1_2020.25555555556'
'USA_CT_Yale_232_2020_USA_B.1_2020.25'
'USA_HI_QDX_164_2020_USA_B.1_2020.19722222222'
'USA_IL_CDC_1732_2020_USA_A.1_2020.17777777778'
'USA_IL_NM0107_2020_USA_B.1_2020.21944444444'
'USA_IL_NM085_2020_USA_B.1_2020.21666666667'
'USA_LA_BIE_096_2020_USA_B.1.p2_2020.26388888889'
'USA_LA_CDC_0499_2020_USA_B.1_2020.19166666667'
'USA_LA_EVTL016_2020_USA_B.1_2020.31666666667'
'USA_LA_EVTL052_2020_USA_B.1_2020.33611111111'
'USA_LA_EVTL078_2020_USA_B.1_2020.34166666667'
'USA_LA_EVTL122_2020_USA_B.1_2020.34722222222'
'USA_LA_SR0155_2020_USA_B.1.p2_2020.25277777778'

'USA_LA_SR0212_2020_USA_B.1.p2_2020.25277777778'
'USA_MA_MGH_00255_2020_USA_B.1_2020.26388888889'
'USA_MA_MGH_00266_2020_USA_B.1_2020.25'
'USA_MA_MGH_00282_2020_USA_B.1_2020.25'
'USA_MA_MGH_00479_2020_USA_B.1_2020.25833333333'
'USA_MA_MGH_00592_2020_USA_B.1_2020.24166666667'
'USA_MD_HP00041_2020_USA_B.1_2020.21666666667'
'USA_MD_HP00107_2020_USA_B.1_2020.22222222222'
'USA_MI_MDHHS_SC20047_2020_USA_B.1_2020.21944444444'
'USA_MI_MDHHS_SC20134_2020_USA_B.1_2020.23055555556'
'USA_MI_MDHHS_SC20173_2020_USA_B.1_2020.21111111111'
'USA_MI_MDHHS_SC20277_2020_USA_B.1_2020.29722222222'
'USA_MI_MDHHS_SC20338_2020_USA_B.1_2020.22777777778'
'USA_MI_MDHHS_SC20581_2020_USA_B.1_2020.33055555556'
'USA_MI_MDHHS_SC20627_2020_USA_B.1_2020.29166666667'
'USA_MI_MDHHS_SC20639_2020_USA_B.1_2020.31388888889'
'USA_MI_MDHHS_SC20647_2020_USA_B.1_2020.31388888889'
'USA_MN2_MDH2_2020_USA_B_2020.18333333333'
'USA_MN47_MDH47_2020_USA_B_2020.2'
'USA_MN_MDH_564_2020_USA_B.1_2020.28611111111'
'USA_MN_MDH_72_2020_USA_B.1_2020.20833333333'
'USA_MN_MDH_91_2020_USA_A.3_2020.20555555556'
'USA_MN_UW251_2020_USA_B.1.p2_2020.2'
'USA_NE_10744_2020_USA_B.1.1_2020.34444444444'
'USA_NM_UNM_00088_2020_USA_B.1_2020.25'
'USA_NM_UNM_00102_2020_USA_B.1_2020.25'
'USA_NM_UNM_00124_2020_USA_B.1_2020.25277777778'
'USA_NM_UNM_00155_2020_USA_B.1_2020.25555555556'
'USA_NY_NYUMC135_2020_USA_B.1_2020.25'
'USA_NY_NYUMC164_2020_USA_B.1_2020.25'
'USA_NY_NYUMC181_2020_USA_B.1_2020.25'
'USA_NY_NYUMC216_2020_USA_B.1.3_2020.25555555556'
'USA_NY_NYUMC219_2020_USA_B.1.3_2020.25555555556'
'USA_NY_NYUMC225_2020_USA_B.1_2020.21388888889'
'USA_NY_NYUMC237_2020_USA_B.1_2020.26111111111'
'USA_NY_NYUMC268_2020_USA_B.2_2020.26388888889'
'USA_NY_NYUMC270_2020_USA_B.1_2020.26388888889'
'USA_NY_NYUMC360_2020_USA_B.1_2020.26944444444'
'USA_NY_NYUMC391_2020_USA_B.1_2020.28333333333'
'USA_NY_NYUMC537_2020_USA_B.1.26_2020.24722222222'
'USA_NY_NYUMC610_2020_USA_B.1.1_2020.25277777778'
'USA_NY_NYUMC662_2020_USA_B.1_2020.21388888889'
'USA_NY_NYUMC668_2020_USA_B.1.1_2020.21111111111'
'USA_NY_NYUMC695_2020_USA_B.1_2020.23611111111'
'USA_NY_NYUMC734_2020_USA_B.1.3_2020.23611111111'
'USA_NY_NYUMC756_2020_USA_B.1_2020.21944444444'
'USA_NY_NYUMC915_2020_USA_B.1_2020.35277777778'
'USA_NY_PV08120_2020_USA_B.1_2020.21111111111'

'USA_NY_PV09141_2020_USA_B.1.3_2020.21944444444'
'USA_NY_PV09328_2020_USA_B.2_2020.21388888889'
'USA_NY_PV09340_2020_USA_B.1_2020.23055555556'
'USA_NY_PV09370_2020_USA_B.1_2020.21666666667'
'USA_NY_PV09371_2020_USA_B.1_2020.21666666667'
'USA_NY_PV09406_2020_USA_B.1_2020.19722222222'
'USA_NY_PV09415_2020_USA_B.1_2020.19722222222'
'USA_NY_PV09478_2020_USA_B.1.1_2020.21944444444'
'USA_NY_WCMP12E06P_2020_USA_B.1_2020.20833333333'
'USA_NY_Wadsworth_10695_01_2020_USA_B.1_2020.175'
'USA_PA_MGSC42_05_2020_USA_B.1_2020.23611111111'
'USA_PA_MGSC49_05_2020_USA_B.1_2020.23611111111'
'USA_TX_HMH0207_2020_USA_A.3_2020.23055555556'
'USA_TX_HMH0397_2020_USA_B.1_2020.25'
'USA_TX_HMH054_2020_USA_B.1_2020.20833333333'
'USA_TX_HMH058_2020_USA_B.1_2020.21111111111'
'USA_TX_HMH06_2020_USA_A.3_2020.20833333333'
'USA_TX_HMH070_2020_USA_B.1_2020.21388888889'
'USA_TX_HMH099_2020_USA_B.1_2020.21666666667'
'USA_UN_UW_2139_2020_USA_B.1_2020.22777777778'
'USA_UT_01578_2020_USA_B.1_2020.26666666667'
'USA_UT_01989_2020_USA_B.1_2020.28888888889'
'USA_UT_03475_2020_USA_B.1_2020.21666666667'
'USA_UT_0503_2020_USA_B.1_2020.25277777778'
'USA_UT_098_2020_USA_B.1_2020.20277777778'
'USA_VA_6171_2020_USA_A.p7_2020.18055555556'
'USA_VA_DCLS_0049_2020_USA_B.1_2020.24722222222'
'USA_VA_DCLS_0200_2020_USA_B.1_2020.27777777778'
'USA_VA_DCLS_0210_2020_USA_B.1_2020.29166666667'
'USA_VA_DCLS_0212_2020_USA_B.1_2020.25833333333'
'USA_VA_DCLS_0229_2020_USA_B.1_2020.26111111111'
'USA_VA_DCLS_0246_2020_USA_B.1_2020.29166666667'
'USA_VA_DCLS_0353_2020_USA_B.1.37_2020.35277777778'
'USA_WA7_UW4_2020_USA_A.1_2020.16666666667'
'USA_WA_NH20_2020_USA_A.1_2020.2'
'USA_WA_S1004_2020_USA_B.1_2020.32777777778'
'USA_WA_S1011_2020_USA_B.1_2020.32777777778'
'USA_WA_S1016_2020_USA_A.1_2020.32777777778'
'USA_WA_S109_2020_USA_A.1_2020.16666666667'
'USA_WA_S1149_2020_USA_B.1_2020.34444444444'
'USA_WA_S1171_2020_USA_B.1.1_2020.34444444444'
'USA_WA_S177_2020_USA_A.1_2020.21666666667'
'USA_WA_S210_2020_USA_A.1_2020.21666666667'
'USA_WA_S222_2020_USA_A.1_2020.21944444444'
'USA_WA_S316_2020_USA_A.1_2020.25'
'USA_WA_S37_2020_USA_A.1_2020.175'
'USA_WA_S437_2020_USA_A.1_2020.25'
'USA_WA_S470_2020_USA_B.1_2020.26111111111'

'USA_WA_S558_2020_USA_B.1_2020.24722222222'
'USA_WA_S598_2020_USA_A.1_2020.28333333333'
'USA_WA_S635_2020_USA_A.1_2020.29444444444'
'USA_WA_S670_2020_USA_B.1_2020.26944444444'
'USA_WA_S718_2020_USA_B.1.43_2020.27777777778'
'USA_WA_S755_2020_USA_A.1_2020.28611111111'
'USA_WA_S757_2020_USA_A.1_2020.28333333333'
'USA_WA_S762_2020_USA_A.1_2020.28611111111'
'USA_WA_S766_2020_USA_B.1_2020.28611111111'
'USA_WA_S767_2020_USA_B.1_2020.28611111111'
'USA_WA_S76_2020_USA_A.1_2020.17777777778'
'USA_WA_S812_2020_USA_A.1_2020.28888888889'
'USA_WA_S872_2020_USA_A.1_2020.31388888889'
'USA_WA_S964_2020_USA_B.1.1_2020.33333333333'
'USA_WA_S9_2020_USA_A.1_2020.16666666667'
'USA_WA_UW111_2020_USA_A.1_2020.19444444444'
'USA_WA_UW139_2020_USA_A.1_2020.19722222222'
'USA_WA_UW152_2020_USA_A.1_2020.2'
'USA_WA_UW205_2020_USA_A.1_2020.2'
'USA_WA_UW214_2020_USA_A.1_2020.20277777778'
'USA_WA_UW325_2020_USA_A.1_2020.20833333333'
'USA_WA_UW_1567_2020_USA_B.1.p21_2020.21666666667'
'USA_WA_UW_1659_2020_USA_B.1.41_2020.21944444444'
'USA_WA_UW_1888_2020_USA_B.1.p21_2020.22777777778'
'USA_WA_UW_2253_2020_USA_B.1_2020.23055555556'
'USA_WA_UW_4109_2020_USA_B.1.p21_2020.25'
'USA_WA_UW_4414_2020_USA_B.1_2020.25'
'USA_WA_UW_5397_2020_USA_B.1.p21_2020.26111111111'
'USA_WA_UW_586_2020_USA_B.1.p2_2020.19722222222'
'USA_WA_UW_6671_2020_USA_B.1_2020.27777777778'
'USA_WI_GMF_00857_2020_USA_B.1_2020.27222222222'
'USA_WI_UW_148_2020_USA_B.1_2020.26666666667'
'USA_WI_UW_226_2020_USA_B.1_2020.23611111111'
'USA_WI_UW_252_2020_USA_A.3_2020.23055555556'
'USA_WI_UW_25_2020_USA_B.2.1_2020.22777777778'
'USA_WI_UW_276_2020_USA_B.1_2020.25'
'USA_WI_UW_335_2020_USA_B.1_2020.23055555556'
'USA_WY_WYPHL_00035_2020_USA_B_2020.23055555556'
'UnitedArabEmirates_L2409_2020_United_Arab_Emirates_B.4_2020.15'
'Wales_PHWC_23C21_2020_United_Kingdom_B.3_2020.20277777778'
'Wales_PHWC_23FA0_2020_United_Kingdom_B.3_2020.20555555556'
'Wales_PHWC_256A9_2020_United_Kingdom_B.1_2020.23055555556'
'Wales_PHWC_25B6E_2020_United_Kingdom_B.2.1_2020.23333333333'
'Wales_PHWC_25BF5_2020_United_Kingdom_B.3_2020.23055555556'
'Wales_PHWC_25C01_2020_United_Kingdom_B.1_2020.23333333333'
'Wales_PHWC_25F08_2020_United_Kingdom_B.3_2020.23611111111'
'Wales_PHWC_266E4_2020_United_Kingdom_B_2020.23888888889'
'Wales_PHWC_26927_2020_United_Kingdom_B.1.1_2020.24166666667'

'Wales_PHWC_26C4C_2020_United_Kingdom_B.1.1.2_2020.24444444444'
'Wales_PHWC_26DEF_2020_United_Kingdom_B.3_2020.24722222222'
'Wales_PHWC_26F9E_2020_United_Kingdom_B.1.67_2020.24444444444'
'Wales_PHWC_273EC_2020_United_Kingdom_B.1.1_2020.24722222222'
'Wales_PHWC_27470_2020_United_Kingdom_B.1.1.p11_2020.25'
'Wales_PHWC_27689_2020_United_Kingdom_B.2.1_2020.25'
'Wales_PHWC_27BB7_2020_United_Kingdom_B.1.1_2020.25'
'Wales_PHWC_27C3C_2020_United_Kingdom_B.1_2020.25'
'Wales_PHWC_285E5_2020_United_Kingdom_B.1_2020.25'
'Wales_PHWC_289BC_2020_United_Kingdom_B.2.2_2020.25555555556'
'Wales_PHWC_28C2C_2020_United_Kingdom_B.1_2020.25'
'Wales_PHWC_28CE0_2020_United_Kingdom_B.3_2020.25277777778'
'Wales_PHWC_2907A_2020_United_Kingdom_B.1.67_2020.25555555556'
'Wales_PHWC_29159_2020_United_Kingdom_B.1_2020.25555555556'
'Wales_PHWC_294BA_2020_United_Kingdom_B.1.1_2020.25'
'Wales_PHWC_29E07_2020_United_Kingdom_B.1_2020.25833333333'
'Wales_PHWC_2A264_2020_United_Kingdom_B.1_2020.26111111111'
'Wales_PHWC_2A817_2020_United_Kingdom_B.2_2020.26666666667'
'Wales_PHWC_2B0D2_2020_United_Kingdom_B.1.1.2_2020.26944444444'
'Wales_PHWC_2B3BB_2020_United_Kingdom_B.1_2020.27777777778'
'Wales_PHWC_2B719_2020_United_Kingdom_B.1_2020.28055555556'
'Wales_PHWC_2B88F_2020_United_Kingdom_B.2_2020.27222222222'
'Wales_PHWC_2BC38_2020_United_Kingdom_B.1.1_2020.27222222222'
'Wales_PHWC_2BE50_2020_United_Kingdom_B.1.1_2020.275'
'Wales_PHWC_2BF20_2020_United_Kingdom_B.1.1_2020.26944444444'
'Wales_PHWC_2BF3F_2020_United_Kingdom_B.1.1.p16_2020.26944444444'
'Wales_PHWC_2D243_2020_United_Kingdom_B.1.1_2020.25277777778'
'Wales_PHWC_2D2F8_2020_United_Kingdom_B.1.1_2020.25833333333'
'Wales_PHWC_2D708_2020_United_Kingdom_B.1_2020.26111111111'
'Wales_PHWC_2E224_2020_United_Kingdom_B.1.1_2020.26666666667'
'Wales_PHWC_2E312_2020_United_Kingdom_B.1.1_2020.29166666667'
'Wales_PHWC_2EAFF_2020_United_Kingdom_B.1.1_2020.26666666667'
'Wales_PHWC_2EB74_2020_United_Kingdom_B.1.1_2020.26388888889'
'Wales_PHWC_2EBA1_2020_United_Kingdom_B.1.p11_2020.26388888889'
'Wales_PHWC_2EFA5_2020_United_Kingdom_B.1.1.2_2020.25833333333'
'Wales_PHWC_2F01A_2020_United_Kingdom_B.1.p11_2020.25833333333'
'Wales_PHWC_2F26F_2020_United_Kingdom_B.1_2020.26388888889'
'Wales_PHWC_30A09_2020_United_Kingdom_B.1_2020.26944444444'
'Wales_PHWC_30CB8_2020_United_Kingdom_B.1.1_2020.27777777778'
'Wales_PHWC_30E58_2020_United_Kingdom_B.1_2020.27222222222'
'Wales_PHWC_317D4_2020_United_Kingdom_B.1.1.p16_2020.28333333333'
'Wales_PHWC_31ABD_2020_United_Kingdom_B.1_2020.27777777778'
'Wales_PHWC_31B23_2020_United_Kingdom_B.1.1_2020.28055555556'
'Wales_PHWC_3207E_2020_United_Kingdom_B.1.1_2020.28333333333'
'Wales_PHWC_33AD9_2020_United_Kingdom_B.1.1_2020.30277777778'
'Wales_PHWC_33B12_2020_United_Kingdom_B.1.1_2020.3'
'Wales_PHWC_3409A_2020_United_Kingdom_B.1_2020.30277777778'
'Wales_PHWC_344AD_2020_United_Kingdom_B.1.1_2020.29722222222'

'Wales_PHWC_34689_2020_United_Kingdom_B.1.1_2020.30277777778'
'Wales_PHWC_348A1_2020_United_Kingdom_B.1_2020.30277777778'
'Wuhan_HBCDC_HB_02_2019_China_B_2019.99722222222'
'Wuhan_HB_WH1_135_2020_China_B_2020.15'
'Wuhan_HB_WH3_172_2020_China_B_2020.16944444444'
'Wuhan_HB_WHCM_103_2020_China_B_2020.16944444444'
'Wuhan_Hu_1_2019_China_B_2019.98611111111'
'Wuhan_IPBCAMS_WH_02_2019_China_B_2019.99722222222'
'Wuhan_WIV07_2019_China_B_2019.99722222222'
