# Supplementary material for: Genomic epidemiology reveals transmission patterns and dynamics of SARS-CoV-2 in Aotearoa New Zealand
Source: Nat Commun. 2020 Dec 11;11:6351. doi: 10.1038/s41467-020-20235-8 (PMC7733492; doi:10.1038/s41467-020-20235-8)
Supplement: Supplementary file 4 — Reporting Summary [file 41467_2020_20235_MOESM4_ESM.pdf]

nature research

Corresponding author(s): Jemma L Geoghegan

Last updated by author(s): Nov 18, 2020

Reporting Summary

Nature Research wishes to improve the reproducibility of the work that we publish. This form provides structure for consistency and transparency in reporting. For further information on Nature Research policies, see our Editorial Policies and the Editorial Policy Checklist.

Statistics

For all statistical analyses, confirm that the following items are present in the figure legend, table legend, main text, or Methods section.

n/a	Confirmed	
☐	☒	The exact sample size (*n*) for each experimental group/condition, given as a discrete number and unit of measurement
☒	☐	A statement on whether measurements were taken from distinct samples or whether the same sample was measured repeatedly
☐	☒	The statistical test(s) used AND whether they are one- or two-sided<br>*Only common tests should be described solely by name; describe more complex techniques in the Methods section.*
☒	☐	A description of all covariates tested
☐	☒	A description of any assumptions or corrections, such as tests of normality and adjustment for multiple comparisons
☐	☒	A full description of the statistical parameters including central tendency (e.g. means) or other basic estimates (e.g. regression coefficient) AND variation (e.g. standard deviation) or associated estimates of uncertainty (e.g. confidence intervals)
☐	☒	For null hypothesis testing, the test statistic (e.g. *F*, *t*, *r*) with confidence intervals, effect sizes, degrees of freedom and *P* value noted<br>*Give P values as exact values whenever suitable.*
☐	☒	For Bayesian analysis, information on the choice of priors and Markov chain Monte Carlo settings
☒	☐	For hierarchical and complex designs, identification of the appropriate level for tests and full reporting of outcomes
☐	☒	Estimates of effect sizes (e.g. Cohen's *d*, Pearson's *r*), indicating how they were calculated

Our web collection on statistics for biologists contains articles on many of the points above.

Software and code

Policy information about availability of computer code

Data collection	N/A
Data analysis	We used open source software for this study: ARTIC bioinformatics medaka pipeline (v 1.1.0), trimmomatic (v 0.36), Burrows-Wheeler Alignment tool (v 1.0), iVar (v 1.2), Picard (v 2.10.10), bcftools mpileup (v 1.9), vcflib (v 1.0.0), MAFFT (v 7), IQ-TREE (v 1.6.8), TempEst (v 1.5.3), pangolin (v 2.0) (https://github.com/hCoV-2019/pangolin), Least Squares Dating (v 1.8), BEAST (v 1.10.4), NELSI (https://github.com/sebastianduchene/NELSI), BEAST (v 2.5), R and Matlab. Phylogenetic tree files and code used to analyse them are available online (https://github.com/sebastianduchene/summarise_importations).

For manuscripts utilizing custom algorithms or software that are central to the research but not yet described in published literature, software must be made available to editors and reviewers. We strongly encourage code deposition in a community repository (e.g. GitHub). See the Nature Research guidelines for submitting code & software for further information.

Data

Policy information about availability of data

All manuscripts must include a data availability statement. This statement should provide the following information, where applicable:

- Accession codes, unique identifiers, or web links for publicly available datasets
- A list of figures that have associated raw data
- A description of any restrictions on data availability

All data is available via open source databases. We have submitted genomic data generated in this study to NCBI under BioProject PRJNA648792. All genetic data are also available via GISAID and accessions are provided in the Supplementary Data File.
These data are plotted in Figures 1-4.
1000 randomly sampled SARS-CoV-2 genomes were obtained from GISAID and the accession numbers of these are provided in a table attached.

Table 1 contains demographic information from 26/02/2020 - 01/07/2020 and is available here https://www.health.govt.nz/our-work/diseases-and-conditions/covid-19-novel-coronavirus/covid-19-current-situation/covid-19-current-cases

Field-specific reporting

Please select the one below that is the best fit for your research. If you are not sure, read the appropriate sections before making your selection.

☒ Life sciences ☐ Behavioural & social sciences ☐ Ecological, evolutionary & environmental sciences

For a reference copy of the document with all sections, see nature.com/documents/nr-reporting-summary-flat.pdf

Life sciences study design

All studies must disclose on these points even when the disclosure is negative.

Sample size	There was a total of 1,178 laboratory-confirmed cases of COVID-19 in New Zealand during the study period. A total of 733 laboratory-confirmed samples of SARS-CoV-2 were received by the authors for whole genome sequencing. Of this, 649 SARS-CoV-2 genomes passed quality control and were used in this study.
Data exclusions	Nucleotide positions that were less than 20x coverage were masked to N in the final consensus genome. Positions with an alternative allele frequency between 20% to 79% were also masked to N. Genomes that contained more than 10% of Ns did not pass quality control and were excluded from the analysis. This exclusion criteria was pre-established.
Replication	All phylogenetic trees and statistical tests were run in duplicate by authors J.L. Geoghegan and S. Duchene with consistent results.
Randomization	Our analysis included 1000 randomly sampled global SARS-CoV-2 genomes. This was only to show on a phylogenetic tree (Figure 2) and these data were not included in any of the formal analysis.
Blinding	Blinding was not relevant to this study since data included de-identified genomes and therefore is not applicable.

Reporting for specific materials, systems and methods

We require information from authors about some types of materials, experimental systems and methods used in many studies. Here, indicate whether each material, system or method listed is relevant to your study. If you are not sure if a list item applies to your research, read the appropriate section before selecting a response.

Materials & experimental systems

n/a	Involved in the study	
☒	☐	Antibodies
☒	☐	Eukaryotic cell lines
☒	☐	Palaeontology and archaeology
☒	☐	Animals and other organisms
☒	☐	Human research participants
☒	☐	Clinical data
☒	☐	Dual use research of concern

Methods

n/a	Involved in the study	
☒	☐	ChIP-seq
☒	☐	Flow cytometry
☒	☐	MRI-based neuroimaging
